# Envelope Stress Activates Expression of the Twin Arginine Translocation (Tat) System in Salmonella

**DOI:** 10.1128/spectrum.01621-22

**Published:** 2022-08-29

**Authors:** Alexandra R. Rogers, Ezekeial E. Turner, Deauna T. Johnson, Jeremy R. Ellermeier

**Affiliations:** a Department of Microbiology and Immunology, Midwestern University, Glendale, Arizona, USA; b College of Graduate Studies, Midwestern University, Glendale, Arizona, USA; South China Sea Institute Of Oceanology

**Keywords:** *Salmonella*, bile, Psp, envelope stress, stress response

## Abstract

The twin arginine translocation system (Tat) is a protein export system that is conserved in bacteria, archaea, and plants. In Gram-negative bacteria, it is required for the export of folded proteins from the cytoplasm to the periplasm. In Salmonella, there are 30 proteins that are predicted substrates of Tat, and among these are enzymes required for anaerobic respiration and peptidoglycan remodeling. We have demonstrated that some conditions that induce bacterial envelope stress activate expression of a Δ*tatABC-lacZ* fusion in Salmonella enterica serovar Typhimurium. Particularly, the addition of bile salts to the growth medium causes a 3-fold induction of a Δ*tatABC-lacZ* reporter fusion. Our data demonstrate that this induction is mediated via the phage shock protein (Psp) stress response system protein PspA. Further, we show that deletion of *tatABC* increases the induction of *tatABC* expression in bile salts. Indeed, the data suggest significant interaction between PspA and the Tat system in the regulatory response to bile salts. Although we have not identified the precise mechanism of Psp regulation of *tatABC*, our work shows that PspA is involved in the activation of *tatABC* expression by bile salts and adds another layer of complexity to the Salmonella response to envelope stress.

**IMPORTANCE** Salmonella species cause an array of diseases in a variety of hosts. This research is significant in showing induction of the Tat system as a defense against periplasmic stress. Understanding the underlying mechanism of this regulation broadens our understanding of the Salmonella stress response, which is critical to the ability of the organism to cause infection.

## INTRODUCTION

The twin arginine translocation (Tat) system is a protein export system present in the cytoplasmic membrane of many bacteria and archaea. In Gram-negative organisms, the Tat translocon transports substrate proteins from the cytoplasm to the periplasmic space where the protein can undergo further export out of the cell or remain in the periplasm. Substrates of Tat are proteins that must be folded in the cytoplasm, often because they contain essential cofactors that are limiting in the periplasmic space (1, 2). This process is independent of ATP and requires the use of proton motive force (PMF) to drive substrate translocation (3, 4). The secretion complex is made of three proteins: TatA or TatE, TatB, and TatC (2, 5, 6). Much work has been done to understand the assembly of the Tat system at the cytoplasmic membrane. These data show that either TatA or TatE are recruited to a TatB/TatC complex bound to a Tat substrate, and TatA or TatE seem to form translocation channels for the substrate (7, 8). TatA or TatE is recruited to the complex in variable numbers to allow for translocation of substrates of various sizes (8). Despite being encoded in a single *tatABC* operon, there are about 25 copies of TatA per TatBC, suggesting posttranscriptional regulation. Indeed, published evidence suggests that this variable number of Tat structural components is due, at least in part, to mRNA decay-based regulation (9). Tat is named after the N-terminal twin arginine signal sequence commonly associated with Tat substrates. Secreted proteins carry the twin arginine signal sequence, (S/T)RRxFLK, which binds to the translocon and initiates movement to the periplasm (1, 10, 11). While loss of the Tat system is lethal in some organisms, it is not lethal to Salmonella enterica serovar Typhimurium (S. Typhimurium); however, there are pleiotropic effects on metabolism, virulence, and proper cell envelope development (6, 12, 13). Tat mutants are also more susceptible than wild-type S. Typhimurium to antimicrobial agents, such as detergents and β-lactam antibiotics (14–17).

The main structural components of the Tat system are encoded in the gene *tatABC* operon (referred to here as the *tat* operon) (18). TatE, which is separately encoded from other Tat complex proteins, is functionally equivalent to TatA (18).

The total number of Tat substrates varies by species. S. Typhimurium has 30 proteins that are either experimentally confirmed or predicted to be exported via Tat (12). These Tat substrates include enzymes necessary for anaerobic respiration, hydrogenases, and cell wall amidases, among other critical cell processes (12). Indeed, the Tat system has been shown to be critical for the virulence of S. Typhimurium and other pathogenic species (12, 15, 19–21) but is not essential for *in vitro* growth. In S. Typhimurium, this virulence defect is due to the combined loss of *amiA*, *amiC*, and *sufI* (12, 21). AmiA and AmiC are *N*-acetylmuramyl-l-alanine amidases that remove cross-links in peptidoglycan during cell division (22, 23), while SufI (FtsP) is important for stabilization of the divisome (22, 24). The combined data from Escherichia coli and S. Typhimurium suggest a very important role of the Tat system in maintenance of the Gram-negative cell envelope. Strains deleted for genes encoding the Tat apparatus experience several growth phenotypes, including elongated cells, septal defects during division, and altered proton motive force (PMF) (17, 22, 25). While little is known about the regulation of *tat* genes, previous work has demonstrated a role for CpxR in directly binding the *tat* promoter and activating *tat* gene expression in response to protamine (26).

Envelope or extracytoplasmic stress responses (ESRs) detect stressors to the envelope and initiate actions to repair or prevent damage. There are several well-studied ESRs identified in S. Typhimurium, including Cpx, Bae, Rcs, σ^E^, and Psp (27–29). Evidence from Escherichia coli is clear that these systems demonstrate a substantial amount of overlap both in what stressors they sense and in what genes they control in response to stress (29). For example, each of the pathways listed have been shown to be induced at least somewhat in response to 4 mM indole, but the Bae response seems to be most dramatic (29). The Cpx system responds to misfolded proteins in the envelope and regulates a wide array of genes, including Tat substrates *amiA* and *amiC* (30). Additionally, CpxR has been shown to directly induce *tatABC* expression (26). The BaeSR two-component system controls a small subset of genes involved in production of efflux pumps (31). The Rcs system is a phosphorelay system that controls colanic acid production and aids in the maintenance of PMF (32). The alternative sigma factor, σ^E^, recruits RNA polymerase to the promoters of genes encoding chaperones, proteases, and outer membrane biogenesis factors needed to respond to envelope stress (33, 34). These stress response systems are reviewed in detail by Macritchie and Raivio (27).

The phage shock protein (Psp) stress response system is a variation on the two-component system, which has multiple resident membrane components (35) and plays a role in maintenance of PMF (36). Primary components of the Psp system include the transcriptional enhancer protein PspF, which interacts with the alternate sigma factor RpoN (σ^54^) to activate transcription of a small subset of the σ^54^ regulon. PspF is only known to directly activate expression of the *pspABCDE* operon *pspF* and *pspG* (29). PspA is an antagonist to PspF, binding PspF so it cannot interact with σ^54^ to activate gene expression (35). PspB and PspC are membrane sensor components that sense mislocalized or misfolded membrane proteins and recruit PspA to the membrane, freeing PspF to activate transcription (37, 38). PspA has several other additional roles in stress response in the cell. In E. coli, it has been shown that PspA is able to form large oligomers to bind areas on the cytoplasmic membrane where there is an increase in stress, and PspA accumulation at the membrane seems to stabilize the membrane to help maintain PMF (39). PspA has recently been described as a member of the endosomal sorting complexes required for transport (ESCRT)-III membrane remodeling family of proteins with homologs to eukaryotes and archaea (40). Several links between the Psp and Tat systems have already been established. TatA and PspA have been demonstrated to interact in the membrane by copurification and electron microscopy (41). This effect is apparently independent of PspF, suggesting a basal level of PspA in the cell (41). Additional work has shown that overproduction of Tat substrates SufI and CueO actually cause inefficient Tat secretion by outcompeting other Tat substrates for use of the Tat machinery, but concurrent overexpression of PspA somehow relieves this saturation and allows more substrates out of the cytoplasm (42). Additionally, previous work has shown that a deletion of *tatC* acts as a stressor on the cell, leading to activation of *psp* gene expression (43).

Given the critical role of Tat in virulence and maintenance of the S. Typhimurium envelope, we sought to determine if there was environmental regulation of the *tat* operon. In this work, we show that some environmental conditions that induce envelope stress activate expression of the *tat* operon in S. Typhimurium. This regulation is dependent on both the Tat structural components and the Psp system, likely through the known interaction of TatA and PspA.

## RESULTS

### Envelope stress conditions activate *tat* expression.

Given the critical nature of the twin arginine translocation (Tat) system in proper cell envelope biogenesis, we predicted that at least some conditions that induce envelope stress in S. Typhimurium would activate expression of *tatABC* to compensate for envelope damage. We constructed a transcriptional Δ*tatABC-lacZ* fusion and monitored expression under different conditions. The data show (Table 1) that 5% and 9% bile salts, 4 mM indole, 0.1% SDS, and 4% ethanol induced expression of Δ*tatABC-lacZ* to various degrees, while 0.5 mM dibucaine, 0.6 mM NaCl, and heat shock at 42°C had no significant impact on *tatABC-lacZ* activity. Given the relatively large 4-fold change in Δ*tatABC-lacZ* expression, we chose to focus primarily on bile salts as a mechanism for studying the induction of *tatABC*. To determine if bile salt activation of Δ*tatABC-lacZ* is physiologically significant, we looked at translocation of the artificial Tat substrate TorA-mCherry-SsrA (44). In this instance, the signal sequence from the Tat substrate TorA is engineered in front of mCherry to translocate the protein to the periplasm via Tat. The SsrA tag leads to degradation of cytoplasmic mCherry; thus, fluorescence indicates translocation of the construct (44). We then assayed for mCherry fluorescence in 0%, 5%, and 9% bile salts. Indeed, the data show that TorA-mCherry-SsrA is translocated to the periplasm at a higher level when cells are grown in bile salts (Fig. S1 in the supplemental material). As expected, deletion of *tatABC* leads to little TorA-mCherry-SsrA translocation and low fluorescence.

**TABLE 1:** Effect of periplasmic stress agents on *tatABC-lacZ* expression

Growth condition	Relative β-galactosidase activity^*a*^	*P* value vs LB control^*b*^
LB control	100.00 ± 4.34	NA
42°C	73.09 ± 4.35	NS
0.6 mM NaCl	86.36 ± 0.81	NS
0.5 mM Dibucaine	99.35 ± 4.79	NS
4% Ethanol	160.84 ± 9.37	0.005
5% Bile salts	174.79 ± 2.61	<0.001
4 mM Indole	177.14 ± 6.78	<0.001
0.10% SDS	180.64 ± 9.70	<0.001
9% Bile salts	397.81 ± 43.87	<0.001

a LB with no additives grown at 37°C set to 100 U of β-galactosidase activity. Strain JRE143 was used under each condition.

b Significance was determined by one-way ANOVA; NS, not significant; NA, not applicable.

**FIG 1:**
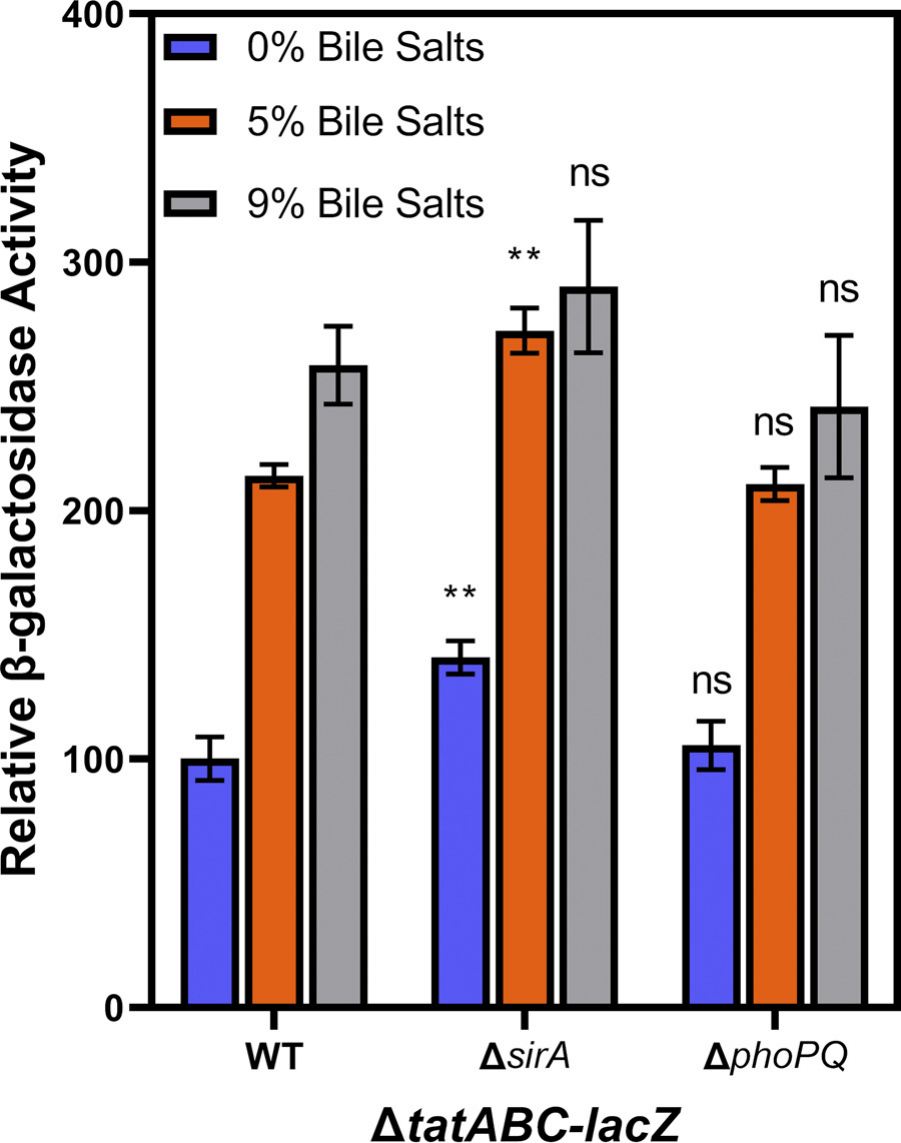
Bile activates expression of *tatABC-lacZ* independently of BarA/SirA and PhoPQ. All strains contain transcriptional *lacZ* fusions to *tatABC* and are otherwise wild-type (WT) or deleted for *sirA* or *phoPQ* as indicated. All strains were grown in LB with 0%, 5%, or 9% bile salts added. LB with no (0%) bile salts was set to 100 U of β-galactosidase activity. Strains used were JRE143, JRE237, and JRE275. Significance was determined by one-way analysis of variance (ANOVA) with strains compared to WT at the same concentration of bile salts; NS, not significant; *, *P* < 0.05; **, *P* < 0.01; ***, *P* < 0.001.

It has been repeatedly shown that pathogenicity of S. Typhimurium is responsive to a variety of environmental signals that alter the expression of genes driving invasion of intestinal epithelial cells (45, 46), survival within macrophages (47, 48), and an array of other critical bacterial processes. S. Typhimurium encounters bile as it transits from the stomach to the small intestine (49), and it plays an important role in the regulatory network of Salmonella (49–51). The BarA/SirA two-component system has been implicated in the S. Typhimurium response to bile (49, 52), although that effect has recently been attributed to other mechanisms (53). To determine if BarA/SirA is involved in bile regulation of *tat*, we moved a *sirA::*cm allele into the Δ*tatABC-lacZ* background and grew the strains in Luria-Bertani (LB) with no additive or 9% bile salts. Bile salts contain roughly equal amounts of sodium cholate and sodium deoxycholate but are missing many components of physiologically produced bile. The data show that disruption of *sirA* has no impact on bile activation of *tat* (Fig. 1). Furthermore, when SirA is overexpressed in the vector pBAD30, it does not activate Δ*tatABC-lacZ* over the vector-only control in any concentration of bile (data not shown). The PhoP/PhoQ two-component system is primarily known for controlling the cellular response to Mg^2+^ and Ca^2+^ ions and antimicrobial peptides (54–56). Additionally, it has been demonstrated that the PhoP/PhoQ system does play a role in the overall cellular bile response in S. Typhimurium (50, 57). Work from the Finlay lab demonstrated that physiological bile induces *tat* expression about 2-fold in a Salmonella enterica serovar Typhi (S. Typhi) microarray, and this is at least partially via PhoPQ (50). Therefore, we moved a deletion of *phoPQ* into the Δ*tatABC-lacZ* construct and monitored *tat* expression in LB with no additive or 9% bile salts. The data show that Δ*phoPQ*::Cm had no impact on *tatABC-lacZ* expression at any level of bile salts (Fig. 1).

### Tat response to bile salts is dependent on the Psp system.

**FIG 2:**
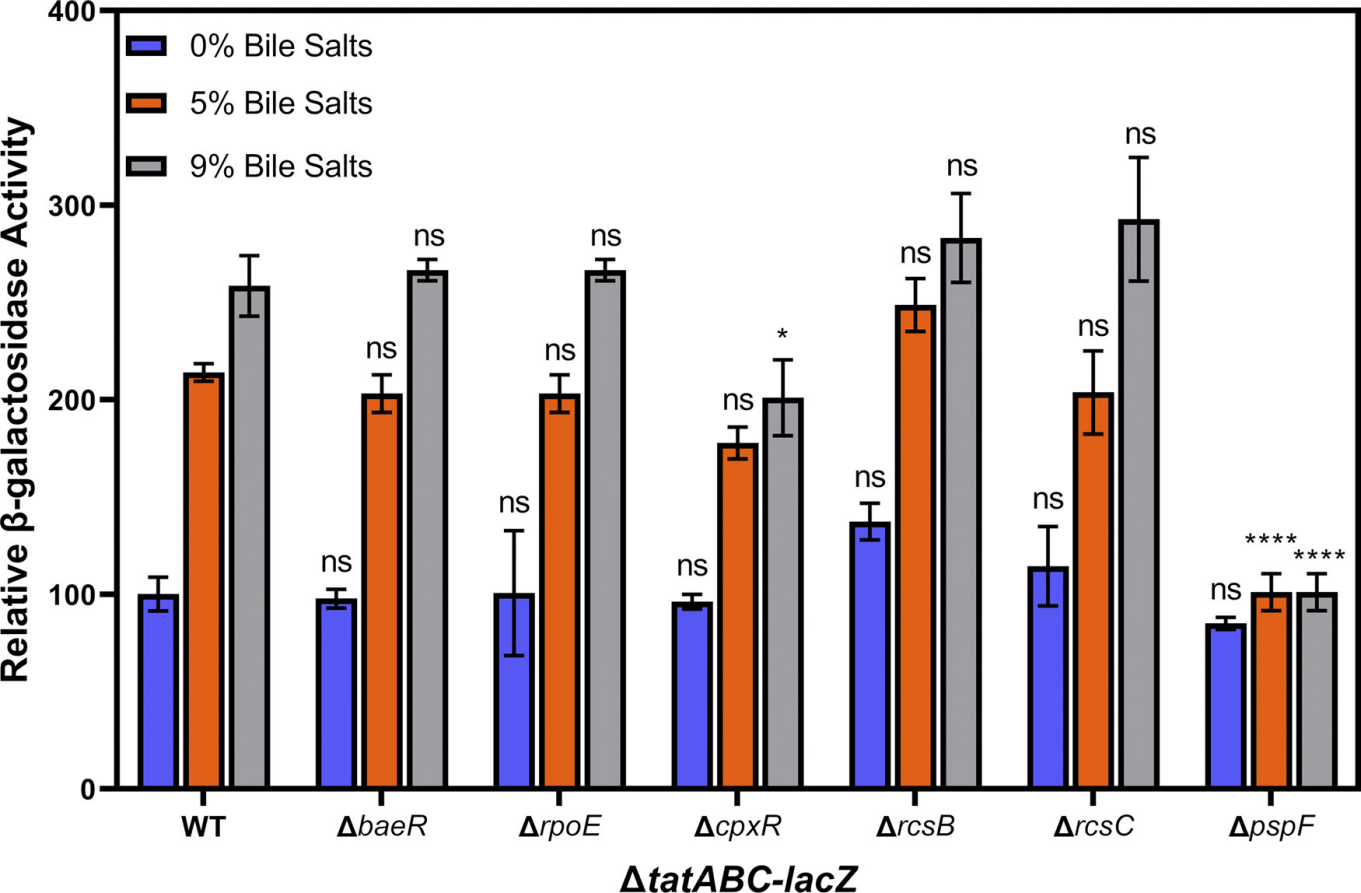
Bile activates expression of *tatABC-lacZ* independently of the σE, Cpx, Rcs, and BaeRS systems but is dependent on Psp. All strains contain transcriptional *lacZ* fusions to *tatABC* and are otherwise wild-type (WT) or are deleted for *rpoE*, *cpxR*, *baeR*, *rcsB*, *rcsC*, or *pspF* as indicated. All strains were grown in LB with 0%, 5%, or 9% bile salts added as indicated. LB with no (0%) bile salts was set to 100 U of β-galactosidase activity. Strains used were JRE143, JRE269, JRE271, JRE273, JRE322, JRE324, and JRE325. Significance was determined by one-way ANOVA with strains compared to WT at the same concentration of bile salts; NS, not significant; *, *P* < 0.05; **, *P* < 0.01; ***, *P* < 0.001.

S. Typhimurium encodes several well-studied systems that respond to stress in the cell envelope, including σ^E^, BaeSR, Cpx, Rcs, and Psp (27). Each of these plays a specific role in response to a set of envelope stressors, although these are often overlapping among the different systems. To test the effect of known stress response systems on *tatABC* expression, we deleted genes associated with each and monitored effects on Δ*tatABC-lacZ* via β-galactosidase assays. We deleted genes associated with the Cpx (*cpxR*), σ^E^ (*rpoE*), BaeSR (*baeR*), and Rcs (*rcsB* and *rcsC*) systems. CpxR is the response regulator in the Cpx system and is phosphorylated by CpxA (27, 29). σ^E^ is an alternative sigma factor that is sequestered at the cytoplasmic membrane by RseA. Envelope stress leads to release of σ^E^ via proteolytic cleavage of RseA by DegS (27, 58). BaeRS is a classic two-component regulatory system with a small regulon. BaeR is the response regulator and is phosphorylated by BaeS when the cell is exposed to toxic compounds (59). RcsA is an auxiliary factor in the Rcs system, as RcsB is the most critical transcriptional activator and can function in the absence of RcsA. RcsC is an unusual sensor kinase that phosphorylates RcsD, which in turn phosphorylates RcsB. Phosphorylated RcsB can form a homodimer or a heterodimer with RcsA. Each complex regulates a subset of the Rcs regulon (27, 32, 60). Single deletions of *rpoE*, *baeR*, *rcsB*, and *rcsC* had no significant impact on bile activation of Δ*tatABC-lacZ* (Fig. 2). CpxR has previously been shown to bind directly to the *tatABC* promoter (26); however, Δ*tatABC-lacZ* shows only a small (1.3-fold), although statistically significant, change in expression in the Δ*cpxR* background (Fig. 2).

**FIG 3:**
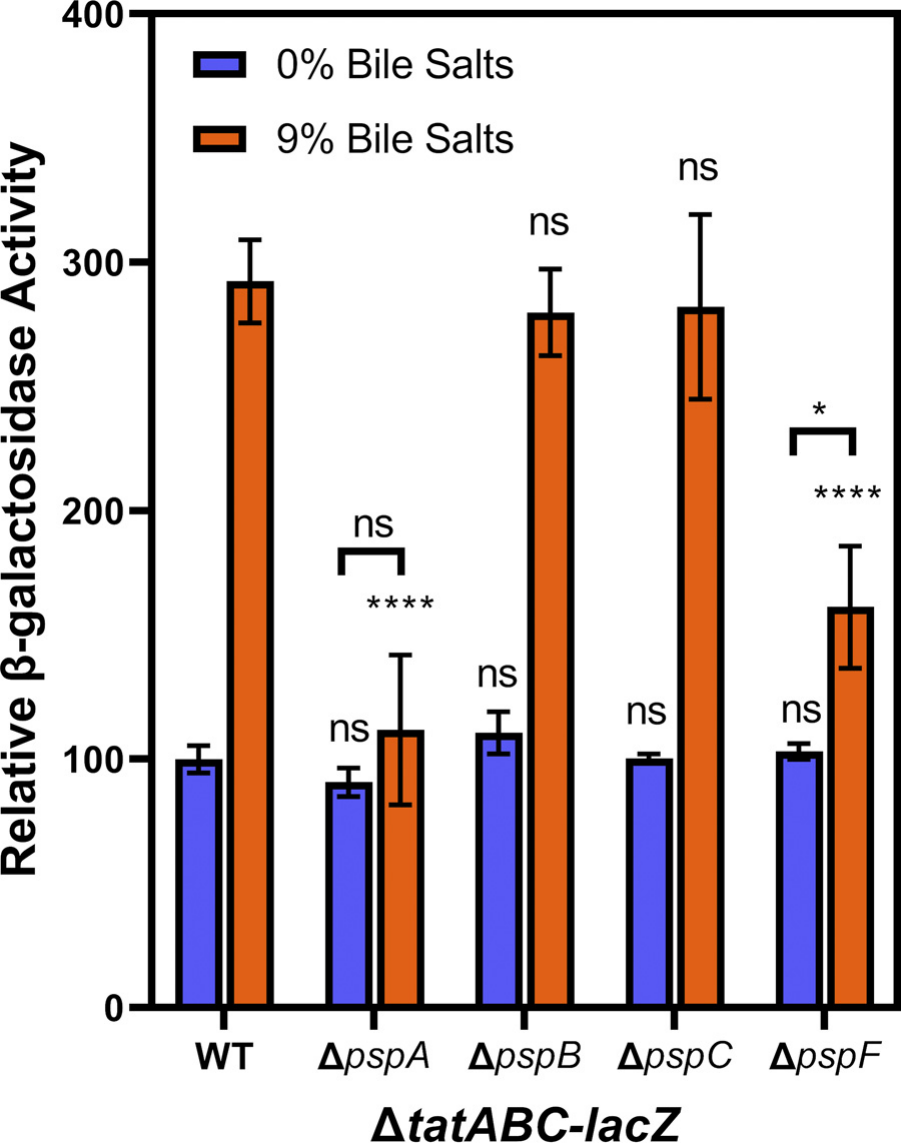
Bile activation of *tatABC-lacZ* expression is dependent on PspA and PspF. All strains contain transcriptional *lacZ* fusions to *tatABC* and are otherwise wild-type (WT) or are deleted for *pspF*, *pspA*, *pspB*, or *pspC* as indicated. All strains were grown in LB with 0% or 9% bile salts added. LB with no (0%) bile salts was set to 100 U of β-galactosidase activity. Strains used were JRE143, JRE 567, JRE322, JRE904, and JRE1033. Significance was determined by one-way ANOVA with strains compared to WT at the same concentration of bile salts; NS, not significant; *, *P* < 0.05; **, *P* < 0.01; ***, *P* < 0.001.

We next deleted components of the Psp system (*pspF*). PspF is a transcriptional regulator that binds to σ^54^ and activates gene expression in response to stress conditions (29, 35). PspA inhibits PspF function by direct protein-protein interaction and is also required for membrane integrity (35, 37). Thus, if the Psp system was activating Δ*tatABC-lacZ*, we would expect the Δ*tatABC-lacZ* fusion to be nonresponsive to bile salts in a Δ*pspF* background. Indeed, the data show that in a Δ*pspF* background, addition of bile salts no longer induces Δ*tatABC-lacZ* expression (Fig. 2), demonstrating that bile salt activation of Δ*tatABC-lacZ* occurs via the Psp system. We further deleted *pspA*, *pspB*, and *pspC* to determine the effect on Δ*tatABC-lacZ*. Given the role of PspA as an antagonist of PspF, we predicted that a deletion of *pspA* would result in an increase in *tatABC* expression. The data show that a *pspA* deletion behaves identically to a deletion of *pspF*, and Δ*tatABC-lacZ* no longer responds to bile salts. Given the polar nature of the Δ*pspA*::Cm allele, we moved deletions of *pspB* and *pspC* into the Δ*tatABC-lacZ* background to confirm that the phenotype is caused by loss of *pspA* and not disruptions to the rest of the downstream operon. The data show that Δ*pspB* and Δ*pspC* have no impact on Δ*tatABC-lacZ* expression, and regulation by bile salts is dependent on PspA (Fig. 3).

**FIG 4:**
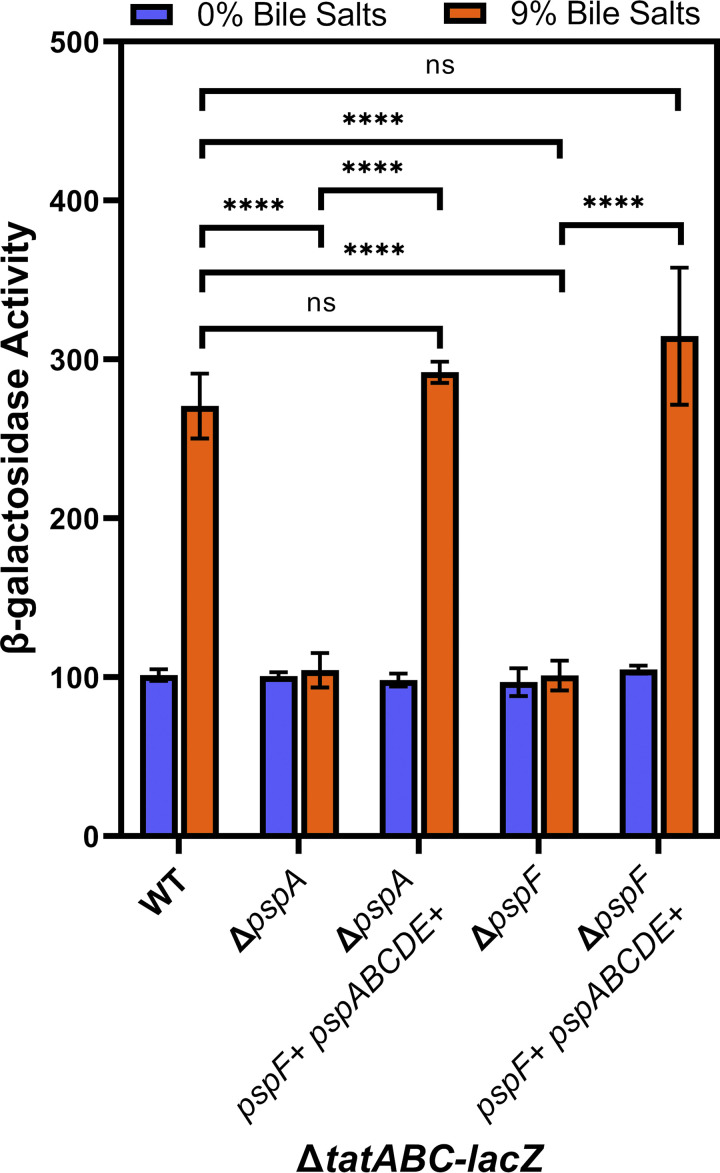
Bile activation of *tatABC-lacZ* is restored with *pspF* and *pspABCDE* complemented in *trans*. All strains contain transcriptional *lacZ* fusions to *tatABC* and are otherwise wild-type (WT) or are deleted for *pspA* or *pspF* as indicated. Strains also contain pJE229 (*psp* region) integrated at the *attB*_λ_ site for complementation of deletions as indicated. All strains were grown in LB with 0% or 9% bile salts added. Strains used were JRE143, JRE567, JRE568, JRE973, and JRE1010. Significance was determined by one-way ANOVA; NS, not significant; *, *P* < 0.05; **, *P* < 0.01; ***, *P* < 0.001.

To confirm the involvement of the Psp system in the regulation of *tatABC*, we cloned the entire *psp* region from *pspF* to *pspE* into pDX1, an apramycin-resistant derivative of the plasmid pAH125. The resulting plasmid (pJE229) was integrated into the Salmonella chromosome at the *attB*_λ_ site (61, 62). In this instance, *psp* genes are all under the control of their native promoters in single copy; therefore, any abnormal effects of PspA overproduction are mitigated while still providing data on complementation. The data show that pJE229 integrated at *attB*_λ_ complements the *pspA* and *pspF* deletions (Fig. 4), confirming that the Psp system plays a role in activation of *tatABC* expression in response to bile salts.

### Deletion of *tatABC* induces *tatABC* expression in high bile salts.

**FIG 5:**
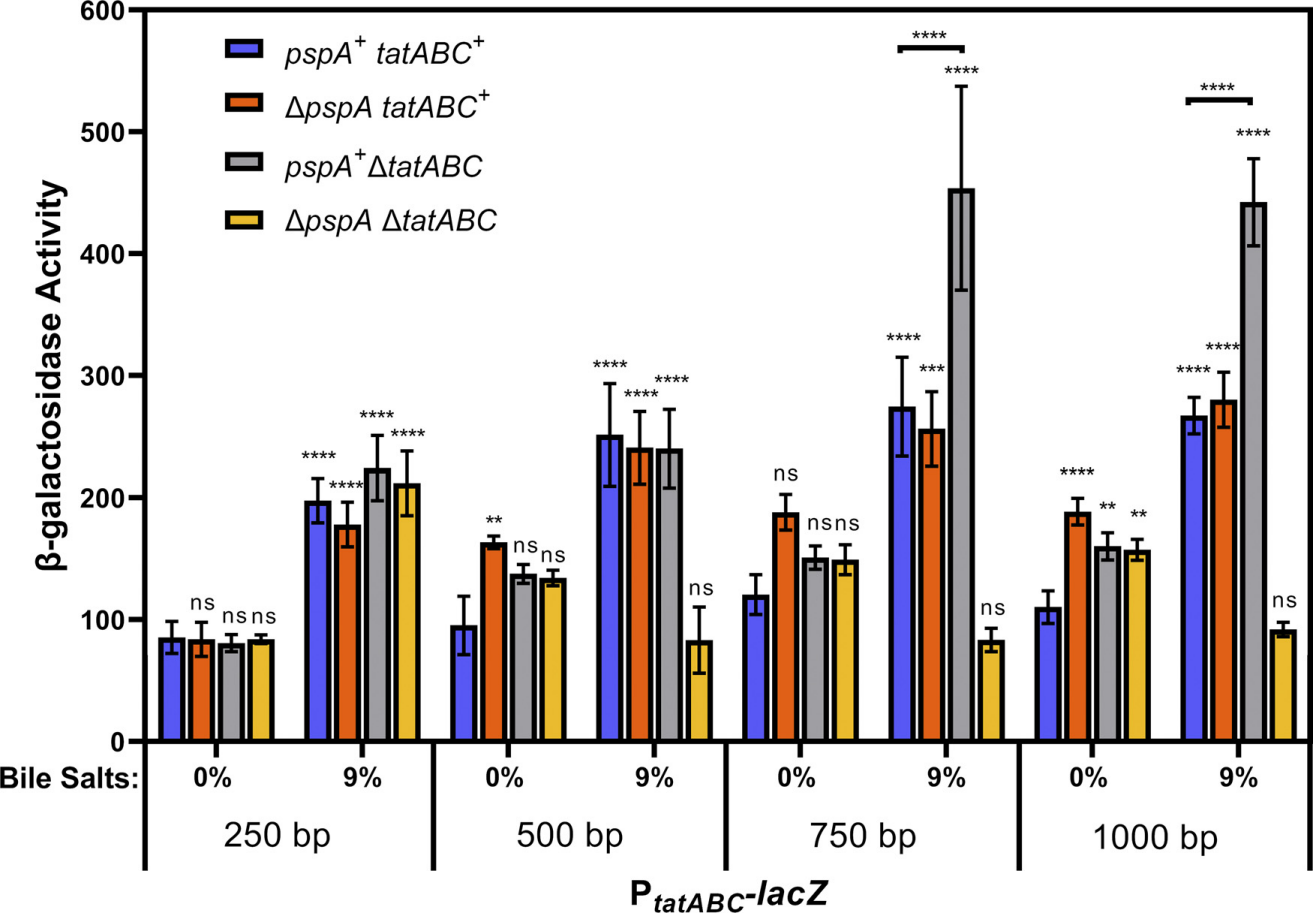
Deletion of *pspA* abrogates induction of *tatABC-lacZ* expression by bile salts in a Δ*tatABC* background. All strains contain transcriptional *lacZ* fusions to different lengths of the *tatABC* promoter integrated at the *attB*_λ_ site. Strains are otherwise wild-type (WT) or are deleted for *pspA*, *tatABC*, or *pspA tatABC*. All strains were grown in LB with 0% or 9% bile salts added. Strains used were JRE651, JRE656, JRE659, JRE661, JRE663, JRE667, JRE701 through JRE705, JRE708, JRE722, JRE735, JRE774, and JRE824. Significance was determined by one-way ANOVA with comparisons made between WT of the same promoter length at 0% bile salts, unless noted; NS, not significant; *, *P* < 0.05; **, *P* < 0.01; ***, *P* < 0.001.

We sought to determine the minimal promoter region necessary for *tatABC* activation by bile salts. To do this, we cloned promoter fragments of various length in front of the promoterless *lacZ* in pAH125 for integration in the S. Typhimurium chromosome at the *attB*_λ_ site and monitored *lacZ* activity via β-galactosidase assays. We cloned fragments of 1,000 bp upstream of the TatA start codon, 750 bp, 500 bp, and 250 bp. Our data demonstrate that there is no difference between the 1,000-bp, 750-bp, and 500-bp fragments in terms of an overall response to bile salts (Fig. 5). There is a slightly lower level of *tatABC* induction in the 250-bp fragment. Additionally, we moved deletions of *pspA* and *tatABC* into the *P_tatABC_*-*lacZ* fusion background. These data show that deletion of *tatABC* causes an induction of *P_tatABC_*-*lacZ* expression in 9% bile salts, and a deletion of both *pspA* and *tatABC* together causes a decrease in the expression of *tatABC* in 9% bile salts. The 1,000-bp, 750-bp, and 500-bp fragments show regulatory effects of deleting *pspA* and *tatABC*. Further, the 250-bp fragment still responds to bile, albeit at a slightly lower level, but does not show a Δ*tatABC* or Δ*pspA* effect. This suggests that disruption to the proper formation of the Tat complex activates expression of *tatABC* genes and this regulatory effect occurs somewhere between 250 bp and 500 bp upstream of the TatA start codon. Published work has shown that CpxR binds directly to *P_tatABC_* at bases 96 to 110 from the TatA start codon (26). Thus, it is possible that the residual effect of bile salts on the 250-bp fragment is due to CpxR regulation. To determine if this is the case, we made targeted mutations to the CpxR binding box on the 250-bp *tatABC* promoter fragment to inhibit CpxR binding and activation (Fig. S2). The data show that the altered 250-bp promoter fragment is still activated by bile salts and is unaffected by deletions of *tatABC* and *pspA* (Fig. S3).

**FIG 6:**
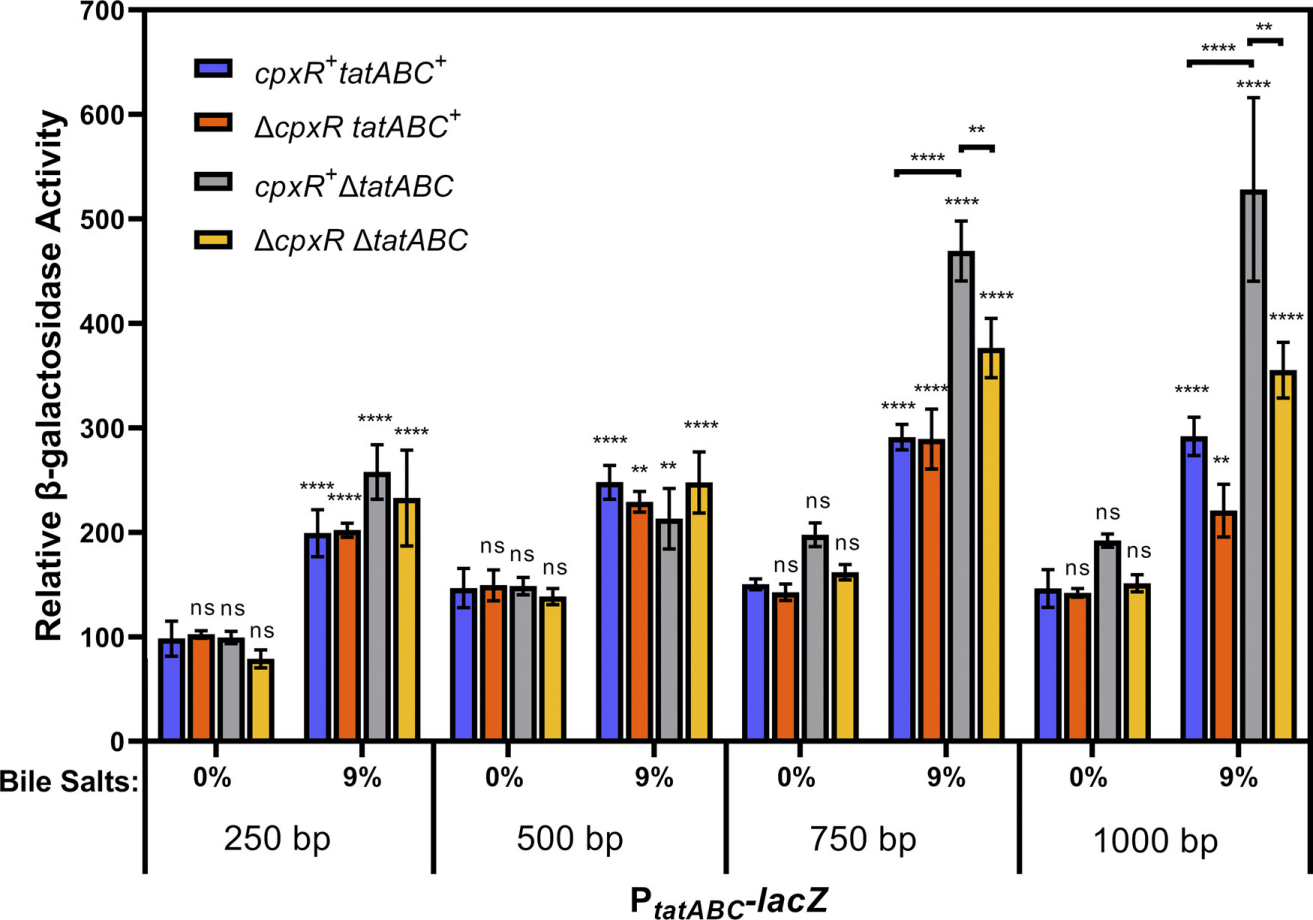
The Cpx system plays a minor role in bile salt activation of *tatABC-lacZ* expression. All strains contain transcriptional *lacZ* fusions to different lengths of the *tatABC* promoter integrated at the *attB*_λ_ site. Strains are otherwise wild-type (WT) or are deleted for *cpxR*, *tatABC*, or *cpxR tatABC*. All strains were grown in LB with 0% or 9% bile salts added. Strains used were JRE651, JRE656, JRE659, JRE661, JRE663, JRE667, JRE722, JRE735, JRE743 through JRE745, JRE775, JRE1031, and JRE1032. Significance was determined by one-way ANOVA with comparisons made between WT of the same promoter length at 0% bile salts, unless noted; NS, not significant; *, *P* < 0.05; **, *P* < 0.01; ***, *P* < 0.001.

Further, we moved a deletion of *cpxR* into the promoter fusion fragments in the *tatABC^+^* and Δ*tatABC* backgrounds. The data show that deletion of *cpxR* has a small but significant impact on the expression of the *P_tatABC_*-*lacZ* fusion in response to bile salts at any length of promoter (Fig. 6); thus, while CpxR binds to the *tatABC* promoter, it seems to have a minor role in the activation of *tatABC* in response to bile salts.

### Overproduction of Tat substrates does not induce *tat* expression.

With the importance of PspA in regulation of the Tat system and the known interaction between PspA and Tat during overproduction of Tat substrates (41, 63), we predicted that overproduction of Tat substrates would induce *tatABC* gene expression as well. To test this, we cloned genes encoding several Tat substrates, *sufI*, *amiA*, *amiC*, *fhuD*, *wcaM*, and *cueO*, into the arabinose inducible vector pBAD33 and assayed expression of the 1,000-bp *P_tatABC_*-*lacZ* fusion in *tatABC*^+^ and Δ*tatABC* backgrounds. The data show that overproduction of the tested substrates had no significant effect on *P_tatABC_*-*lacZ* expression over the vector control in either the *tatABC*^+^ or the Δ*tatABC* backgrounds (Table 2). Given these results, we did not pursue the cloning of other Tat substrates.

**TABLE 2:** Effect of Tat substrate overproduction on *P_tatABC_*-*lacZ* expression

	Relative β-galactosidase activity			
Plasmid^*a*^	*tatABC^+^*	*P* value^*b*^ vs pBAD33 *tatABC^+^*	Δ*tatABC*	*P* value^*b*^ vs pBAD33 *tatABC^+^*
pBAD33	100.00 ± 5.36	NA	103.86 ± 2.21	NS
pJE216 (AmiA)	96.11 ± 7.06	NS	99.21 ± 3.29	NS
pJE217 (AmiC)	94.18 ± 2.84	NS	101.38 ± 2.53	NS
pJE218 (SufI)	97.87 ± 2.93	NS	114.01 ± 9.67	NS
pJE234 (CueO)	86.21 ± 4.59	NS	104.42 ± 2.34	NS
pJE235 (FhuD)	112.03 ± 3.84	NS	82.07 ± 6.57	NS
pJE236 (WcaM)	96.15 ± 6.31	NS	92.05 ± 6.71	NS

a Overproduction of Tat substrates does not induce *P_tatABC_*-*lacZ* expression. All strains contain transcriptional *lacZ* fusions to the 1,000-bp *tatABC* promoter integrated at the *attB*_λ_ site and indicated plasmids, which are all derived from pBAD33. Strains are otherwise wild-type (WT) or are deleted for *tatABC* as indicated. All strains were grown in LB with chloramphenicol included for plasmid maintenance and 0.2% arabinose included for induction of the *P_BAD_* promoter. Strains used were JRE1070 through JRE1081, JRE1101, and JRE1102.

b Significance was determined by one-way ANOVA; NS, not significant; NA, not applicable.

## DISCUSSION

The Tat system is required for proper maintenance of the bacterial cell envelope; thus, proper expression of the components of the Tat translocon is critical to the bacterium (12, 16). Our data demonstrate that some conditions, including high concentrations of bile salts, that stress the Salmonella envelope also activate *tatABC* gene expression. This activation seems to be dependent on the presence of both TatABC structural proteins and the phage shock protein PspA. β-Galactosidase assays on a S. Typhimurium strain with a *lacZ* reporter fusion to the *tatABC* promoter indicate that bile salts, indole, and SDS are compounds that activate *tatABC* expression. The strongest *tatABC*-activating condition that we have identified is 9% bile salts (Table 1). Craig et al. demonstrated that deletion of *tatC* dramatically attenuates S. Typhimurium virulence in mice and that this is due to the loss of three critical proteins involved in cell wall biogenesis during cell division: AmiA, AmiC, and SufI (12). Additionally, *in vitro* evidence demonstrates that deletions of *tat* are highly susceptible to cell wall-damaging agents, such as bile, SDS, and β-lactam antibiotics (16, 17). Thus, it makes sense that bile would induce expression of *tatABC*, as more substrates of the Tat apparatus are needed to manage potential damage in the cell envelope. Indeed, we also show that increasing bile salts in the growth medium also increases translocation of the artificial Tat substrate TorA-mCherry-SsrA, indicating that a 3-fold increase in transcription of *tatABC* has a significant impact on the amount of Tat substrate translocated (Fig. S1 in the supplemental material). It has been previously shown that the Cpx stress response system activates known Tat substrates *amiA* and *amiC* (30) and binds the *tatABC* promoter (26), although Cpx has only a minor role in the activation of *tatABC* in response to bile salts (Fig. 2 and 6). Additionally, the virulence phenotype of an Δ*amiA* Δ*amiC* strain was attributed to the growth defect in deoxycholate (21).

These data are supported by microarray experiments in S. Typhi, showing that physiological bile purified from mice works partially via the PhoPQ regulatory system (50). Data from Antunes et al. (50) indicate that physiological bile induces *tatABC* expression about 2-fold, although our data show that the PhoPQ system is not involved in activation of Δ*tatABC-lacZ* by bile salts (Fig. 1). The BarA/SirA two-component regulatory system has also been previously implicated in the Salmonella bile response (64, 65); however, our data demonstrate that a *sirA*-null mutant has no impact on *tat* expression (Fig. 1). The PhoP/PhoQ two-component regulatory system had an impact in the bile regulation of Salmonella Typhi (50); however, deleting *phoPQ* had no impact on *tatABC-lacZ* expression in Salmonella Typhimurium. It should be noted that the study by Antunes et al. used physiological bile, and our study uses bile salts; thus, it is possible that some component of physiological bile that is not present in bile salts is responsible for the PhoP/PhoQ effect observed by Antunes et al. (50).

**FIG 7:**
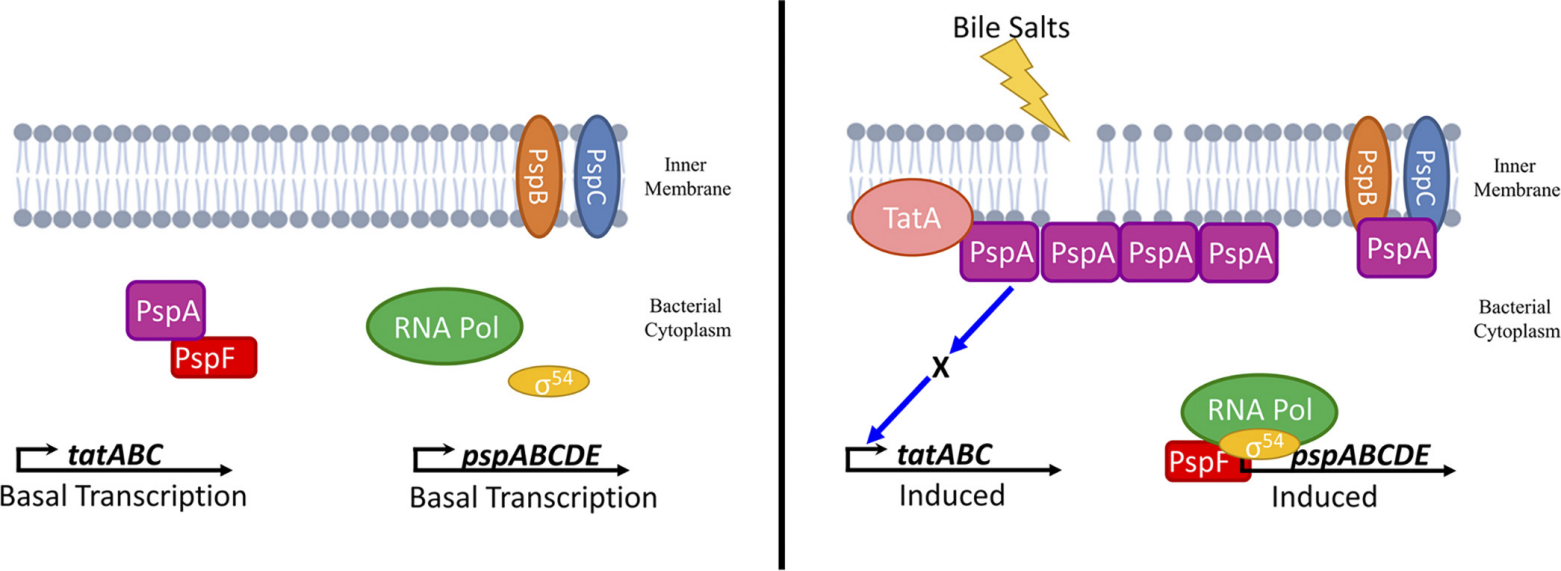
Proposed model of Psp regulation of *tatABC-lacZ* in response to bile salts. Under low bile salt conditions, PspA antagonizes PspF to prevent transcription of the *psp* operon. Under high bile salt conditions, the membrane is stressed, and PspB and PspC recruit PspA to the membrane, freeing PspF to activate the *psp* operon. Accumulation of PspA at the membrane induces expression of *tatABC* via an unknown mechanism; RNA Pol, RNA polymerase.

We constructed Δ*tatABC-lacZ* strains with deletions of five well-studied stress response systems in S. Typhimurium: σ^E^, BaeSR, CpxRA, RcsBCD, and Psp (29). Given the established binding of CpxR to the *tatABC* promoter, we predicted Cpx to be the most likely candidate for regulation of *tatABC* in response to bile salts. Our data indicate that the Cpx system has a small but significant effect on *tatABC* activation by bile salts (Fig. 2 and 6). This small effect on *tatABC-lacZ* expression could account for the small residual induction in the Δ*pspF* background (Fig. 2 and 3). Unfortunately, it is difficult to create a *cpxR pspA tat* deletion strain, as this is lethal in even low concentrations of bile salts. In the 250-bp fragment of the *tatABC* promoter, we see induction in response to bile salts but no effect of deleting either *tatABC* or *pspA* (Fig. 5). We hypothesized that if we mutate the known CpxR binding site in the 250-bp *tatABC* promoter fragment then CpxR would no longer bind, and induction by bile salts would be abrogated. However, in the mutated 250-bp fragment (Fig. S2), we saw no effect of the altered CpxR binding site on regulation, and bile salts still induced the *lacZ* fusion (Fig. S3). This suggests a possible secondary mechanism for regulation of *tatABC* that is independent of the Psp and Cpx systems. Additionally, σ^E^, BaeSR, and Rcs do not play any significant role in bile salt activation of *tatABC* expression (Fig. 2). The data show that the Psp system is required for the activation of *tatABC* in response to bile salts (Fig. 2 and 3). PspF is an enhancer for σ^54^ binding to promoters it controls, and our current understanding suggests that the Psp regulon is limited, controlling the *pspABCDE* operon and *pspG* (35). We confirmed the role of Psp in Δ*tatABC-lacZ* regulation by complementation of the *pspA*-deletion mutant with *pspF* and *pspABCDE* cloned onto pDX1 and integrated into the S. Typhimurium chromosome (Fig. 4). We do not have any data that suggest that PspF regulates *tatABC* outside its normal role in controlling production of PspA. PspA seems to be the critical player in bile salt activation of *tatABC*. Indeed, our model (Fig. 7) proposes that high levels of PspA are leading to induction of *tatABC*.

Data from DeLisa et al. has previously demonstrated a critical role for PspA in Tat substrate translocation, somehow easing the burden on the system when Tat substrates CueO and SufI are overproduced (63). We predicted that overproduction of Tat substrates would induce expression of the *tat* operon via PspA. Our data demonstrate that overproduction of Tat substrates has no significant impact on *tatABC-lacZ* expression (Table 2). Additionally, our data show that deletion of *tatABC* genes themselves causes an increase in *tatABC* expression in response to the addition of bile salts and that the effect of Δ*pspA* only occurs in the Δ*tatABC* background, suggesting that there is a critical mechanistic relationship between Psp and Tat. There is no evidence that PspA is a transcriptional regulator outside its antagonism of PspF, and the structure of PspA does not contain any likely DNA-binding motifs (66); thus, action at the level of *tat* transcription is very likely indirect. The mechanism of action behind this regulation is currently unknown; however, the data provide compelling evidence that the Psp system has an important role in the transcription of *tatABC*. The relationship between the Tat and Psp systems, and that of TatA and PspA in particular, plays an important role in Salmonella physiology, and this work adds another layer to this complex relationship.

Given the apparent functional overlap between TatA and TatE (18), it would also be interesting to see if *tatE*, which is encoded independently of the *tatABC* operon, is activated by any of the same signals as *tatABC*. It is possible that *tatE* expression is tied to other conditions. Although there is still much work to be done to understand the specific mechanisms of how envelope stress is inducing expression of the *tat* operon, we provide evidence of transcriptional regulation of this critical protein translocation system and demonstrate a role for the Psp system in this transcriptional activation.

## MATERIALS AND METHODS

### Media, reagents, and enzymatic assays.

Luria-Bertani (LB) medium was used in all experiments for growth of bacteria, and super optimal broth with catabolite repression (SOC) was used for the recovery of transformants (67), unless otherwise noted. Bacterial strains were routinely grown at 37°C except for strains containing the temperature-sensitive plasmids pINT-ts, pCP20, or pKD46, which were grown at 30°C. Antibiotics were used at the following concentrations: 50 μg/mL ampicillin (Amp), 20 μg/mL chloramphenicol (Cm), 50 μg/mL kanamycin (Km), and 50 μg/mL apramycin (Apr). Enzymes were purchased from New England Biolabs (Ipswich, MA) and were used according to the manufacturer’s recommendations. Primers were purchased from Integrated DNA Technologies (Coralville, IA). Bile salts number 3 and other reagents were purchased from Research Products International (Mt. Prospect, IL). Antibiotics were purchased from MilliporeSigma. (St. Louis, MO). β-Galactosidase assays were performed using a microtiter plate assay as previously described on strains grown under the indicated conditions (68, 69). β-Galactosidase activity units are defined as (micromoles of ortho-nitrophenol (ONP) formed min^−1^) × 10^6^/(optical density at 600 nm [OD_600_] × milliliters of cell suspension) and are reported as mean ± standard deviation, where *n* = 4. Cultures used in the β-galactosidase assay were initially inoculated into LB with no additives and grown for 16 h. Cultures were then subcultured 1:100 in either LB or LB with indicated additive and grown for 5 h at 37°C in a shaking incubator at 225 rpm. Heat-shocked cultures were grown for 5 h at 42°C.

### Strain and plasmid construction.

Bacterial strains and plasmids are described in Table S1 in the supplemental material. All Salmonella enterica serovar Typhimurium strains used in this study are isogenic derivatives of strain 14028 (American Type Culture Collection) and were constructed using P22 HT105/1 *int*-201 (P22)-mediated transduction (67). Deletion of various genes and concomitant insertion of an antibiotic resistance cassette were performed using Lambda Red-mediated recombination as previously described (70). In all cases, the appropriate insertion of the antibiotic resistance marker was checked by P22 linkage to known markers and/or PCR analysis. The constructs resulting from this procedure were moved into a clean, wild-type background (14028) by P22 transduction. In some strains, the antibiotic resistance cassettes were removed using the temperature-sensitive plasmid pCP20 carrying FLP recombinase (71). For construction of the Δ*tatABC-lacZ* reporter fusion strain, a kanamycin-resistant deletion of *tatABC* was generated using primers P106 and P107 (Table S2). The insertion mutation generated by Lambda Red-mediated recombination was converted to transcriptional *lacZ* fusions using an FLP/FLP recombination target (FRT)-mediated site-specific recombination method, as previously described (72). Plasmids constructed in this work were verified by sequencing analysis at the Arizona State University Genomics Facility. Primers used for the deletions and cloning are described in Table S2. Plasmid pJE229 was constructed by amplification of the entire region from *pspF* to *pspE* using primers P395 and P396 (Table S2). The amplified product and pDX1 were digested with SphI and NheI. This digest removes *lacZ* while maintaining *oriRg*, apramycin resistance, and *attP*. The pDX1 backbone was gel purified and ligated to the *psp* region and transformed into electrocompetent *pir^+^* E. coli. Different lengths of the *tatABC* promoter were cloned 5′ to the promoterless *lacZ* gene in pAH125 (see Table S2 for detailed primer information) to generate *tatABC*+ *P_tatABC_*-*lacZ* constructs. After sequence verification, all of the resulting pAH125- or pDX1-derived plasmids were integrated into the S. Typhimurium chromosome at the *attB*_λ_ site using λInt produced from the conditional replication, integration, and modular (CRIM) helper plasmid pINT-ts (61). The integrated plasmids were tested by PCR to confirm that a single copy was integrated. pAT6::Cm was made by amplifying *cat* from pBAD33 and digesting the PCR product and pAT6 with PvuI to remove β-lactamase. Digested pAT6 was treated with rSAP to prevent self-ligation, purified, and ligated to the *cat* cassette.

### TorA-mCherry-ssrA translocation assay.

Overnight cultures were subcultured 1:100 in LB with ampicillin and 0.1% arabinose and grown for 5 h at 37°C; 1:100 subcultures were made again in LB with ampicillin and 0.1% arabinose in a 96-well plate. Cells were grown for 14 h at 37°C with shaking and continuous orbital rotation at 180 rpm in a Biotek Cytation 3 plate reader. Every 15 min, cells were excited at 570 nm, and emission was measured and recorded at 610 nm. At each time point, the OD_600_ was also recorded. The following formula was used to calculate normalized mCherry values: (mCherry – mCherry background)/OD_600_. Because bile salts also emit a fluorescent signal at 610 nm, mCherry background was calculated by reading the media-only control wells and taking the mean fluorescence at 610 nm at each of the bile concentrations. The corrected mCherry signal was then divided by OD_600_ to generate a relative fluorescent mCherry signal in each well.

## References

[1] Palmer T, Sargent F, Berks BC. 2005. Export of complex cofactor-containing proteins by the bacterial Tat pathway. Trends Microbiol 13:175–180. doi:10.1016/j.tim.2005.02.002. 15817387

[2] Palmer T, Berks BC. 2012. The twin-arginine translocation (Tat) protein export pathway. Nat Rev Microbiol 10:483–496. doi:10.1038/nrmicro2814. 22683878

[3] Yahr TL, Wickner WT. 2001. Functional reconstitution of bacterial Tat translocation *in vitro*. EMBO J 20:2472–2479. doi:10.1093/emboj/20.10.2472. 11350936 PMC125449

[4] Alami M, Trescher D, Wu L-F, Müller M. 2002. Separate analysis of twin-arginine translocation (Tat)-specific membrane binding and translocation in *Escherichia coli*. J Biol Chem 277:20499–20503. doi:10.1074/jbc.M201711200. 11923313

[5] Orriss GL, Tarry MJ, Ize B, Sargent F, Lea SM, Palmer T, Berks BC. 2007. TatBC, TatB, and TatC form structurally autonomous units within the twin arginine protein transport system of *Escherichia coli*. FEBS Lett 581:4091–4097. doi:10.1016/j.febslet.2007.07.044. 17686475 PMC2517984

[6] Berks BC, Palmer T, Sargent F. 2003. The Tat protein translocation pathway and its role in microbial physiology. Adv Microb Physiol 47:187–254. doi:10.1016/s0065-2911(03)47004-5. 14560665

[7] Gohlke U, Pullan L, McDevitt CA, Porcelli I, de Leeuw E, Palmer T, Saibil HR, Berks BC. 2005. The TatA component of the twin-arginine protein transport system forms channel complexes of variable diameter. Proc Natl Acad Sci USA 102:10482–10486. doi:10.1073/pnas.0503558102. 16027357 PMC1180781

[8] Leake MC, Greene NP, Godun RM, Granjon T, Buchanan G, Chen S, Berry RM, Palmer T, Berks BC. 2008. Variable stoichiometry of the TatA component of the twin-arginine protein transport system observed by in vivo single-molecule imaging. Proc Natl Acad Sci USA 105:15376–15381. doi:10.1073/pnas.0806338105. 18832162 PMC2563114

[9] Dar D, Sorek R. 2018. Extensive reshaping of bacterial operons by programmed mRNA decay. PLoS Genet 14:e1007354. doi:10.1371/journal.pgen.1007354. 29668692 PMC5927463

[10] Berks BC, Sargent F, Palmer T. 2000. The Tat protein export pathway. Mol Microbiol 35:260–274. doi:10.1046/j.1365-2958.2000.01719.x. 10652088

[11] Berks BC. 1996. A common export pathway for proteins binding complex redox cofactors? Mol Microbiol 22:393–404. doi:10.1046/j.1365-2958.1996.00114.x. 8939424

[12] Craig M, Sadik AY, Golubeva YA, Tidhar A, Slauch JM. 2013. Twin-arginine translocation system (*tat*) mutants of *Salmonella* are attenuated due to envelope defects, not respiratory defects. Mol Microbiol 89:887–902. doi:10.1111/mmi.12318. 23822642 PMC3811912

[13] Rollauer SE, Tarry MJ, Graham JE, Jääskeläinen M, Jäger F, Johnson S, Krehenbrink M, Liu S-M, Lukey MJ, Marcoux J, McDowell MA, Rodriguez F, Roversi P, Stansfeld PJ, Robinson CV, Sansom MSP, Palmer T, Högbom M, Berks BC, Lea SM. 2012. Structure of the TatC core of the twin-arginine protein transport system. Nature 492:210–214. doi:10.1038/nature11683. 23201679 PMC3573685

[14] Pradel N, Delmas J, Wu LF, Santini CL, Bonnet R. 2009. Sec- and Tat-dependent translocation of β-lactamases across the *Escherichia coli* inner membrane. Antimicrob Agents Chemother 53:242–248. doi:10.1128/AAC.00642-08. 18981261 PMC2612164

[15] Reynolds MM, Bogomolnaya L, Guo J, Aldrich L, Bokhari D, Santiviago CA, McClelland M, Andrews-Polymenis H. 2011. Abrogation of the twin arginine transport system in *Salmonella enterica* serovar Typhimurium leads to colonization defects during infection. PLoS One 6:e15800. doi:10.1371/journal.pone.0015800. 21298091 PMC3027627

[16] Ize B, Stanley NR, Buchanan G, Palmer T. 2003. Role of the *Escherichia coli* Tat pathway in outer membrane integrity. Mol Microbiol 48:1183–1193. doi:10.1046/j.1365-2958.2003.03504.x. 12787348

[17] Brauer AM, Rogers AR, Ellermeier JR. 2021. Twin-arginine translocation (Tat) mutants in *Salmonella enterica* serovar Typhimurium have increased susceptibility to cell wall targeting antibiotics. FEMS Microbes 2:xtab004. doi:10.1093/femsmc/xtab004. 34250488 PMC8262268

[18] Jack RL, Sargent F, Berks BC, Sawers G, Palmer T. 2001. Constitutive expression of *Escherichia coli tat* genes indicates an important role for the twin-arginine translocase during aerobic and anaerobic growth. J Bacteriol 183:1801–1804. doi:10.1128/JB.183.5.1801-1804.2001. 11160116 PMC95070

[19] Lavander M, Ericsson SK, Bröms JE, Forsberg Å. 2006. The twin arginine translocation system is essential for virulence of *Yersinia pseudotuberculosis*. Infect Immun 74:1768–1776. doi:10.1128/IAI.74.3.1768-1776.2006. 16495550 PMC1418654

[20] Caldelari I, Mann S, Crooks C, Palmer T. 2006. The Tat pathway of the plant pathogen *Pseudomonas syringae* is required for optimal virulence. Mol Plant Microbe Interact 19:200–212. doi:10.1094/MPMI-19-0200. 16529382

[21] Fujimoto M, Goto R, Hirota R, Ito M, Haneda T, Okada N, Miki T. 2018. Tat-exported peptidoglycan amidase-dependent cell division contributes to *Salmonella* Typhimurium fitness in the inflamed gut. PLoS Pathog 14:e1007391. doi:10.1371/journal.ppat.1007391. 30379938 PMC6231687

[22] Heidrich C, Templin MF, Ursinus A, Merdanovic M, Berger J, Schwarz H, de Pedro MA, Höltje JV. 2001. Involvement of *N*-acetylmuramyl-l-alanine amidases in cell separation and antibiotic-induced autolysis of *Escherichia coli*. Mol Microbiol 41:167–178. doi:10.1046/j.1365-2958.2001.02499.x. 11454209

[23] Bernhardt TG, de Boer PAJ. 2003. The *Escherichia coli* amidase AmiC is a periplasmic septal ring component exported via the twin-arginine transport pathway. Mol Microbiol 48:1171–1182. doi:10.1046/j.1365-2958.2003.03511.x. 12787347 PMC4428285

[24] Tarry M, Arends SJR, Roversi P, Piette E, Sargent F, Berks BC, Weiss DS, Lea SM. 2009. The *Escherichia coli* cell division protein and model Tat substrate SufI (FtsP) localizes to the septal ring and has a multicopper oxidase-like structure. J Mol Biol 386:504–519. doi:10.1016/j.jmb.2008.12.043. 19135451 PMC2661564

[25] Samaluru H, Saisree L, Reddy M. 2007. Role of SufI (FtsP) in cell division of *Escherichia coli*: evidence for its involvement in stabilizing the assembly of the divisome. J Bacteriol 189:8044–8052. doi:10.1128/JB.00773-07. 17766410 PMC2168700

[26] Subramaniam S, Müller VS, Hering NA, Mollenkopf H, Becker D, Heroven AK, Dersch P, Pohlmann A, Tedin K, Porwollik S, McClelland M, Meyer TF, Hunke S. 2019. Contribution of the Cpx envelope stress system to metabolism and virulence regulation in *Salmonella enterica* serovar Typhimurium. PLoS One 14:e0211584. doi:10.1371/journal.pone.0211584. 30716090 PMC6361445

[27] Macritchie DM, Raivio TL. 2009. Envelope stress responses. EcoSal Plus doi:10.1128/ecosalplus.5.4.7. 26443759

[28] Grabowicz M, Silhavy TJ. 2017. Envelope stress responses: an interconnected safety net. Trends Biochem Sci 42:232–242. doi:10.1016/j.tibs.2016.10.002. 27839654 PMC5336467

[29] Bury-Moné S, Nomane Y, Reymond N, Barbet R, Jacquet E, Imbeaud S, Jacq A, Bouloc P. 2009. Global analysis of extracytoplasmic stress signaling in *Escherichia coli*. PLoS Genet 5:e1000651. doi:10.1371/journal.pgen.1000651. 19763168 PMC2731931

[30] Weatherspoon-Griffin N, Zhao G, Kong W, Kong Y, Andrews-Polymenis H, McClelland M, Shi Y. 2011. The CpxR/CpxA two-component system up-regulates two tat-dependent peptidoglycan amidases to confer bacterial resistance to antimicrobial peptide. J Biol Chem 286:5529–5539. doi:10.1074/jbc.M110.200352. 21149452 PMC3037666

[31] Leblanc SKD, Oates CW, Raivio TL. 2011. Characterization of the induction and cellular role of the BaeSR two-component envelope stress response of *Escherichia coli*. J Bacteriol 193:3367–3375. doi:10.1128/JB.01534-10. 21515766 PMC3133272

[32] Pando JM, Karlinsey JE, Lara JC, Libby SJ, Fang FC. 2017. The Rcs-regulated colanic acid capsule maintains membrane potential in *Salmonella enterica* serovar Typhimurium. mBio 8:e00808-17. doi:10.1128/mBio.00808-17. 28588134 PMC5461412

[33] Rhodius VA, Suh WC, Nonaka G, West J, Gross CA. 2006. Conserved and variable functions of the σ^E^ stress response in related genomes. PLoS Biol 4:e2. doi:10.1371/journal.pbio.0040002. 16336047 PMC1312014

[34] Skovierova H, Rowley G, Rezuchova B, Homerova D, Lewis C, Roberts M, Kormanec J. 2006. Identification of the σE regulon of *Salmonella enterica* serovar Typhimurium. Microbiology (Reading) 152:1347–1359. doi:10.1099/mic.0.28744-0. 16622052

[35] Joly N, Engl C, Jovanovic G, Huvet M, Toni T, Sheng X, Stumpf MPH, Buck M. 2010. Managing membrane stress: the phage shock protein (Psp) response, from molecular mechanisms to physiology. FEMS Microbiol Rev 34:797–827. doi:10.1111/j.1574-6976.2010.00240.x. 20636484

[36] Becker LA, Bang I-S, Crouch M-L, Fang FC. 2005. Compensatory role of PspA, a member of the phage shock protein operon, in *rpoE* mutant *Salmonella enterica* serovar Typhimurium. Mol Microbiol 56:1004–1016. doi:10.1111/j.1365-2958.2005.04604.x. 15853886

[37] Flores-Kim J, Darwin AJ. 2015. Activity of a bacterial cell envelope stress response is controlled by the interaction of a protein binding domain with different partners. J Biol Chem 290:11417–11430. doi:10.1074/jbc.M114.614107. 25802329 PMC4416846

[38] Jones SE, Lloyd LJ, Tan KK, Buck M. 2003. Secretion defects that activate the phage shock response of *Escherichia coli*. J Bacteriol 185:6707–6711. doi:10.1128/JB.185.22.6707-6711.2003. 14594846 PMC262093

[39] McDonald C, Jovanovic G, Ces O, Buck M. 2015. Membrane stored curvature elastic stress modulates recruitment of maintenance proteins PspA and Vipp1. mBio 6:e01188-15. doi:10.1128/mBio.01188-15. 26330516 PMC4556811

[40] Liu J, Tassinari M, Souza DP, Naskar S, Noel JK, Bohuszewicz O, Buck M, Williams TA, Baum B, Low HH. 2021. Bacterial Vipp1 and PspA are members of the ancient ESCRT-III membrane-remodeling superfamily. Cell 184:3660–3673. doi:10.1016/j.cell.2021.05.041. 34166615 PMC8281802

[41] Mehner D, Osadnik H, Lünsdorf H, Brüser T. 2012. The Tat system for membrane translocation of folded proteins recruits the membrane-stabilizing Psp machinery in *Escherichia coli*. J Biol Chem 287:27834–27842. doi:10.1074/jbc.M112.374983. 22689583 PMC3431631

[42] Tullman-Ercek D, DeLisa MP, Kawarasaki Y, Iranpour P, Ribnicky B, Palmer T, Georgiou G. 2007. Export pathway selectivity of *Escherichia coli* twin arginine translocation signal peptides. J Biol Chem 282:8309–8316. doi:10.1074/jbc.M610507200. 17218314 PMC2730154

[43] Ize B, Porcelli I, Lucchini S, Hinton JC, Berks BC, Palmer T. 2004. Novel phenotypes of *Escherichia coli tat* mutants revealed by global gene expression and phenotypic analysis. J Biol Chem 279:47543–47554. doi:10.1074/jbc.M406910200. 15347649

[44] Bageshwar UK, VerPlank L, Baker D, Dong W, Hamsanathan S, Whitaker N, Sacchettini JC, Musser SM. 2016. High throughput screen for *Escherichia coli* twin arginine translocation (Tat) inhibitors. PLoS One 11:e0149659. doi:10.1371/journal.pone.0149659. 26901445 PMC4764201

[45] Golubeva YA, Sadik AY, Ellermeier JR, Slauch JM. 2012. Integrating global regulatory input into the *Salmonella* pathogenicity Island 1 type III secretion system. Genetics 190:79–90. doi:10.1534/genetics.111.132779. 22021388 PMC3249375

[46] Ellermeier JR, Slauch JM. 2007. Adaptation to the host environment: regulation of the SPI1 type III secretion system in *Salmonella enterica* serovar Typhimurium. Curr Opin Microbiol 10:24–29. doi:10.1016/j.mib.2006.12.002. 17208038

[47] Deiwick J, Nikolaus T, Erdogan S, Hensel M. 1999. Environmental regulation of *Salmonella* pathogenicity island 2 gene expression. Mol Microbiol 31:1759–1773. doi:10.1046/j.1365-2958.1999.01312.x. 10209748

[48] Fass E, Groisman EA. 2009. Control of *Salmonella* pathogenicity island-2 gene expression. Curr Opin Microbiol 12:199–204. doi:10.1016/j.mib.2009.01.004. 19264535 PMC2805070

[49] Prouty AM, Brodsky IE, Falkow S, Gunn JS. 2004. Bile-salt-mediated induction of antimicrobial and bile resistance in *Salmonella typhimurium*. Microbiology (Reading) 150:775–783. doi:10.1099/mic.0.26769-0. 15073288

[50] Antunes LCM, Wang M, Andersen SK, Ferreira RBR, Kappelhoff R, Han J, Borchers CH, Finlay BB. 2012. Repression of *Salmonella enterica phoP* expression by small molecules from physiological bile. J Bacteriol 194:2286–2296. doi:10.1128/JB.00104-12. 22366421 PMC3347055

[51] Nikaido E, Yamaguchi A, Nishino K. 2008. AcrAB multidrug efflux pump regulation in *Salmonella enterica* serovar Typhimurium by RamA in response to environmental signals. J Biol Chem 283:24245–24253. doi:10.1074/jbc.M804544200. 18577510 PMC2527123

[52] Prouty AM, Brodsky IE, Manos J, Belas R, Falkow S, Gunn JS. 2004. Transcriptional regulation of *Salmonella enterica* serovar Typhimurium genes by bile. FEMS Immunol Med Microbiol 41:177–185. doi:10.1016/j.femsim.2004.03.002. 15145463

[53] Eade CR, Hung C-C, Bullard B, Gonzalez-Escobedo G, Gunn JS, Altier C. 2016. Bile acids function synergistically to repress invasion gene expression in *Salmonella* by destabilizing the invasion regulator HilD. Infect Immun 84:2198–2208. doi:10.1128/IAI.00177-16. 27185788 PMC4962646

[54] Navarre WW, Halsey TA, Walthers D, Frye J, McClelland M, Potter JL, Kenney LJ, Gunn JS, Fang FC, Libby SJ. 2005. Co-regulation of *Salmonella enterica* genes required for virulence and resistance to antimicrobial peptides by SlyA and PhoP/PhoQ. Mol Microbiol 56:492–508. doi:10.1111/j.1365-2958.2005.04553.x. 15813739

[55] García Véscovi E, Soncini FC, Groisman EA. 1996. Mg^2+^ as an extracellular signal: environmental regulation of *Salmonella* virulence. Cell 84:165–174. doi:10.1016/s0092-8674(00)81003-x. 8548821

[56] Shi Y, Cromie MJ, Hsu F-F, Turk J, Groisman EA. 2004. PhoP-regulated *Salmonella* resistance to the antimicrobial peptides magainin 2 and polymyxin B. Mol Microbiol 53:229–241. doi:10.1111/j.1365-2958.2004.04107.x. 15225317

[57] van Velkinburgh JC, Gunn JS. 1999. PhoP-PhoQ-regulated loci are required for enhanced bile resistance in *Salmonella* spp. Infect Immun 67:1614–1622. doi:10.1128/IAI.67.4.1614-1622.1999. 10084994 PMC96504

[58] McMeechan A, Roberts M, Cogan TA, Jørgensen F, Stevenson A, Lewis C, Rowley G, Humphrey TJ. 2007. Role of the alternative sigma factors σ^E^ and σ^S^ in survival of *Salmonella enterica* serovar Typhimurium during starvation, refrigeration and osmotic shock. Microbiology 153:263–269. doi:10.1099/mic.0.29235-0. 17185555

[59] Appia-Ayme C, Patrick E, Sullivan MJ, Alston MJ, Field SJ, AbuOun M, Anjum MF, Rowley G. 2011. Novel inducers of the envelope stress response BaeSR in *Salmonella* Typhimurium: baeR is critically required for tungstate waste disposal. PLoS One 6:e23713. doi:10.1371/journal.pone.0023713. 21886814 PMC3160322

[60] Wang Q, Zhao Y, McClelland M, Harshey RM. 2007. The RcsCDB signaling system and swarming motility in *Salmonella enterica* serovar Typhimurium: dual regulation of flagellar and SPI-2 virulence genes. J Bacteriol 189:8447–8457. doi:10.1128/JB.01198-07. 17905992 PMC2168921

[61] Haldimann A, Wanner BL. 2001. Conditional-replication, integration, excision, and retrieval plasmid-host systems for gene structure-function studies of bacteria. J Bacteriol 183:6384–6393. doi:10.1128/JB.183.21.6384-6393.2001. 11591683 PMC100134

[62] Ellermeier JR, Slauch JM. 2008. Fur regulates expression of the *Salmonella* pathogenicity island 1 type III secretion system through HilD. J Bacteriol 190:476–486. doi:10.1128/JB.00926-07. 17993530 PMC2223717

[63] DeLisa MP, Lee P, Palmer T, Georgiou G. 2004. Phage shock protein PspA of *Escherichia coli* relieves saturation of protein export via the Tat pathway. J Bacteriol 186:366–373. doi:10.1128/JB.186.2.366-373.2004. 14702305 PMC305757

[64] Ahmer BM, van Reeuwijk J, Watson PR, Wallis TS, Heffron F. 1999. *Salmonella* SirA is a global regulator of genes mediating enteropathogenesis. Mol Microbiol 31:971–982. doi:10.1046/j.1365-2958.1999.01244.x. 10048039

[65] Teplitski M, Goodier RI, Ahmer BMM. 2003. Pathways leading from BarA/SirA to motility and virulence gene expression in *Salmonella*. J Bacteriol 185:7257–7265. doi:10.1128/JB.185.24.7257-7265.2003. 14645287 PMC296259

[66] Dworkin J, Jovanovic G, Model P. 2000. The PspA protein of *Escherichia coli* is a negative regulator of σ^54^-dependent transcription. J Bacteriol 182:311–319. doi:10.1128/JB.182.2.311-319.2000. 10629175 PMC94278

[67] Maloy S, Stewart V, Taylor R, Miller S. 1996. Genetic analysis of pathogenic bacteria. Cold Spring Harbor Laboratory Press, New York, NY.

[68] Slauch JM, Silhavy TJ. 1991. Genetic fusions as experimental tools. Methods Enzymol 204:213–248. doi:10.1016/0076-6879(91)04011-c. 1658562

[69] Slauch JM, Silhavy TJ. 1991. *cis*-Acting *ompF* mutations that result in OmpR-dependent constitutive expression. J Bacteriol 173:4039–4048. doi:10.1128/jb.173.13.4039-4048.1991. 1648075 PMC208052

[70] Datsenko KA, Wanner BL. 2000. One-step inactivation of chromosomal genes in *Escherichia coli* K-12 using PCR products. Proc Natl Acad Sci USA 97:6640–6645. doi:10.1073/pnas.120163297. 10829079 PMC18686

[71] Cherepanov PP, Wackernagel W. 1995. Gene disruption in *Escherichia coli*: TcR and KmR cassettes with the option of Flp-catalyzed excision of the antibiotic-resistance determinant. Gene 158:9–14. doi:10.1016/0378-1119(95)00193-a. 7789817

[72] Ellermeier CD, Janakiraman A, Slauch JM. 2002. Construction of targeted single copy *lac* fusions using λ Red and FLP-mediated site-specific recombination in bacteria. Gene 290:153–161. doi:10.1016/s0378-1119(02)00551-6. 12062810

