# Supplementary material for: Envelope Stress Activates Expression of the Twin Arginine Translocation (Tat) System in Salmonella
Source: Microbiol Spectr. 2022 Aug 29;10(5):e01621-22. doi: 10.1128/spectrum.01621-22 (PMC9604234; doi:10.1128/spectrum.01621-22)
Supplement: Supplemental file 1 — Supplemental material. Download spectrum.01621-22-s0001.pdf, PDF file, 0.2 MB [file spectrum.01621-22-s0001.pdf]

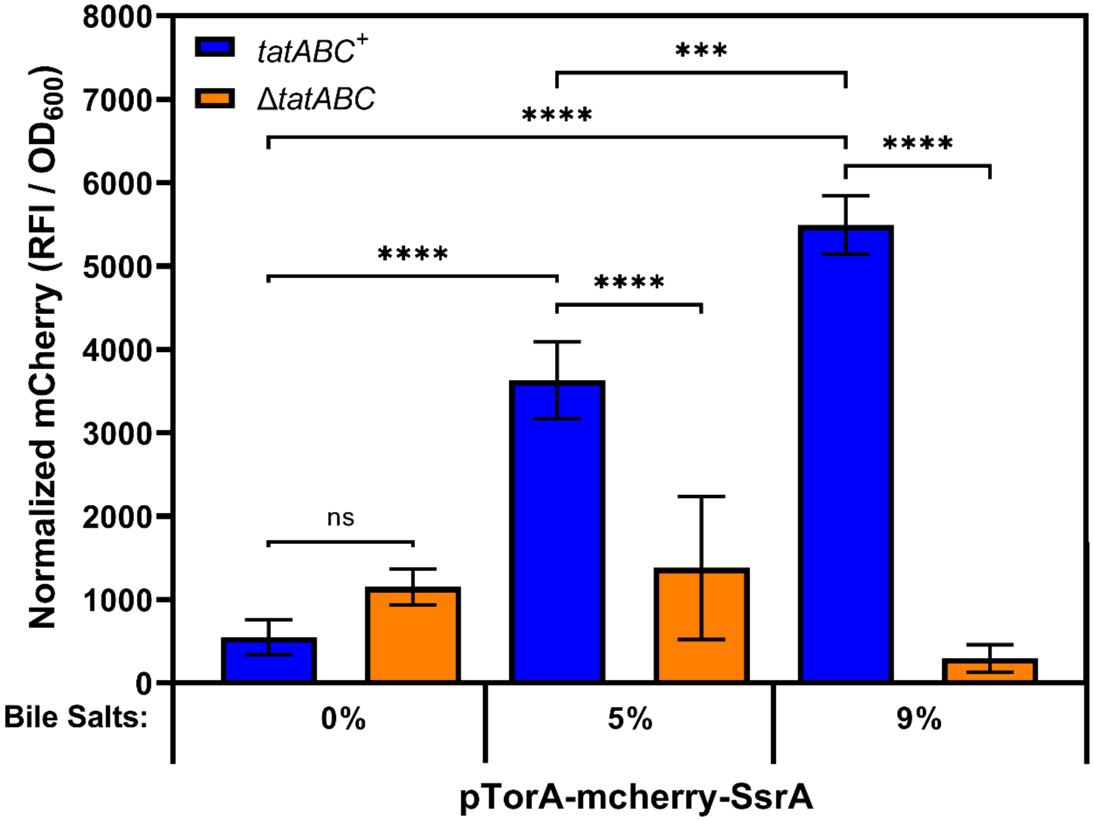

Figure S1. Bile salts increase translocation of the artificial Tat substrate TorA-mCherry-SsrA. All strains contain the artificial Tat substrate TorA-mCherry-SsrA expressed from pBAD30 and are either otherwise wild type (*tatABC*$^{+}$) or deleted for *tatABC* as indicated. All strains were grown in LB with ampicillin and 0%, 5%, or 9% bile salts were added as indicated. Strains used were JRE1066 and JRE 1067.

WT 250 bp pro: GGTCGGCGGCGTAGTTGTCTGGCTGGTTGGCTGGCGTAAAACTCGTTGAGATTTTTTATCG
CpxR binding mut: GGTCGGCGGCGTAGTTGTCTGGCTGGTTGGCTGGCGTAAAACTCGTTGAGATTTTTTATCG

WT 250 bp pro: CTCAACGCCGGTTGTGTCACGCAGGTACATATTATCACTCGAGGCAATACTCAGGCCGCAAG
CpxR binding mut: CTCAACGCCGGTTGTGTCACGCAGGTACATATTATCACTCGAGGCAATACTCAGGCCGCAAG

CpxR Binding

WT 250 bp pro: TCAATGTCGTCCCGGTCGTATGTAAAAGTATGTGAATAGGGCGGGCGAAAGCGGCTAACAA
CpxR binding mut: TCAATGTCGTCCCGGTCGTATGTCGATGTCAGCGCCTAGGGCGGGCGAAAGCGGCTAACAA

WT 250 bp pro: AGAGGCAGCGTGAAGGATAATGTGTATAATGCGGCCCTAATAATTCATCATCTATCACAGAG
CpxR binding mut: AGAGGCAGCGTGAAGGATAATGTGTATAATGCGGCCCTAATAATTCATCATCTATCACAGAG

WT 250 bp pro: GAACATGT
CpxR binding mut: GAACATGT

Figure S2. Alignment of wild type *tatABC* 250 bp promoter fragment with the 250 bp fragment CpxR binding mutant. The CpxR binding site is outlined in red and base changes are indicated by yellow highlight.

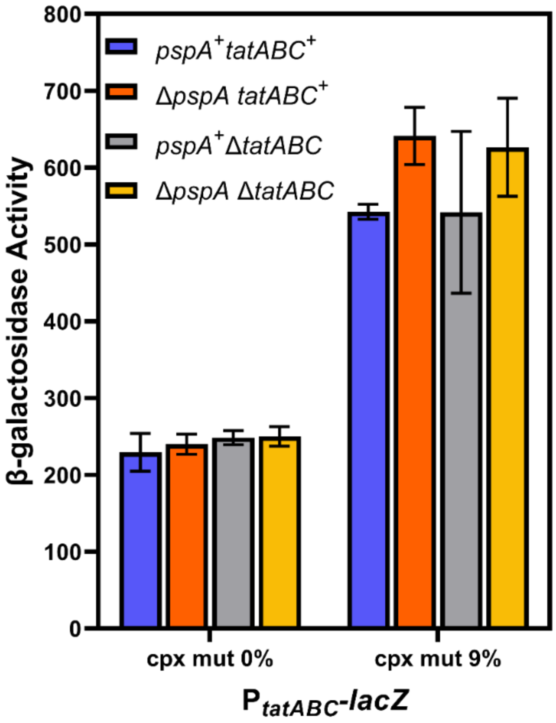

Figure S3. Mutation of the CpxR binding site in the 250 bp *tatABC* promoter fragment does not alter the induction of *tatABC-lacZ* expression. All strains contain transcriptional *lacZ* fusions to *tatABC* and are either otherwise wild type (WT) or are deleted for *tatABC* or *pspA* as indicated. Strains used were JRE857 through JRE860.

Table S1. Bacterial strains and plasmids used in this study.

Strain	Genotype[a]	Source or Reference[b]
14028	Wild Type *Salmonella enterica* serovar Typhimurium	ATCC[c]
	Δ*pspB*::Cm	BEI Resources
	Δ*pspC*::Cm	BEI Resources
BA746	*sirA3*::Cm	(1)
JS1068	Δ*phoPQ*::Cm	(2)
QW434	Δ*rcsB*	(3)
QW398	Δ*rcsC*	(3)
JRE140	Δ*tatABC11*::Kn	(4)
JRE142	Δ*tatABC11*	
JS327	Δ*cpxR100*::Cm	(5)
JRE266	Δ*baeR110*::Cm	
JS2073	Δ*rpoE*::Cm	(6)
JRE315	Δ*pspA162*::Cm	
JRE316	Δ*pspF161*::Cm	
JRE143	Φ(*tatABC-lac')*	
JRE275	Δ*phoPQ*::Cm Φ(*tatABC-lac')*	
JRE273	Δ*cpxR100*::Cm Φ(*tatABC-lac')*	
JRE237	*sirA3*::Cm Φ(*tatABC-lac')*	
JRE269	Δ*baeR110*::Cm Φ(*tatABC-lac')*	
JRE271	Δ*rpoE*::Cm Φ(*tatABC-lac')*	
JRE321	Δ*pspA162*::Cm Φ(*tatABC-lac')*	
JRE567	Δ*pspA162 Φ(tatABC-lac')*	
JRE322	Δ*pspF161*::Cm Φ(*tatABC-lac')*	
JRE239	pBA324 Φ(*tatABC-lac')*	
JRE238	pBAD30 Φ(*tatABC-lac')*	
JRE324	Δ*rcsB* Φ(*tatABC-lac')*	
JRE325	Δ*rcsC* Φ(*tatABC-lac')*	
JRE561	pBAD33 Φ(*tatABC-lac')*	
JRE535	pJE206 Δ*pspA162*::Cm Φ(*tatABC-lac')*	
JRE538	pJE206 Δ*pspF161*::Cm Φ(*tatABC-lac')*	
JRE533	pJE207 Φ(*tatABC-lac')*	
JRE536	pJE207 Δ*pspA162*::Cm Φ(*tatABC-lac')*	
JRE539	pJE207 Δ*pspF161*::Cm Φ(*tatABC-lac')*	
JRE534	pBAD30 Δ*pspA162*::Cm Φ(*tatABC-lac')*	
JRE537	pBAD30 Δ*pspF161*::Cm Φ(*tatABC-lac')*	
JRE904	Δ*pspB*::Cm Φ(*tatABC-lac')*	
JRE1033	Δ*pspC*::Cm Φ(*tatABC-lac')*	
JRE1058	*att*λ::pJE229	
JRE1059	*att*λ::pJE229 Φ(*tatABC-lac')*	
JRE973	*att*λ::pJE229 Δ*pspA162*::Cm Φ(*tatABC-lac')*	
JRE651	*att*λ::pAH125::$P_{tatABC\ 1\text{-}254}$-*lac*$^{+}$ [d]	
JRE722	*att*λ::pAH125::$P_{tatABC\ 1\text{-}517}$-*lac*$^{+}$ [d]	
JRE656	*att*λ::pAH125::$P_{tatABC\ 1\text{-}769}$-*lac*$^{+}$ [d]	
JRE659	*att*λ::pAH125::$P_{tatABC\ 1\text{-}991}$-*lac*$^{+}$ [d]	
JRE701	Δ*pspA162*::Cm *att*λ::pAH125::$P_{tatABC\ 1\text{-}254}$-*lac*$^{+}$ [d]	
JRE774	Δ*pspA162*::Cm *att*λ::pAH125::$P_{tatABC\ 1\text{-}517}$-*lac*$^{+}$ [d]	
JRE703	Δ*pspA162*::Cm *att*λ::pAH125::$P_{tatABC\ 1\text{-}769}$-*lac*$^{+}$ [d]	
JRE705	Δ*pspA162*::Cm *att*λ::pAH125::$P_{tatABC\ 1\text{-}991}$-*lac*$^{+}$ [d]	
JRE661	Δ*tatABC11 att*λ::pAH125::$P_{tatABC\ 1\text{-}254}$-*lac*$^{+}$ [d]	
JRE735	Δ*tatABC11 att*λ::pAH125::$P_{tatABC\ 1\text{-}517}$-*lac*$^{+}$ [d]	

JRE663	Δ*tatABC11* *att*λ::pAH125::$P_{tatABC\ 1\text{-}769}$-*lac*$^{+}$ [d]
JRE667	Δ*tatABC11* *att*λ::pAH125::$P_{tatABC\ 1\text{-}991}$-*lac*$^{+}$ [d]
JRE702	Δ*tatABC11* Δ*pspA162*::Cm *att*λ::pAH125::$P_{tatABC\ 1\text{-}254}$-*lac*$^{+}$ [d]
JRE824	Δ*tatABC11* Δ*pspA162*::Cm *att*λ::pAH125::$P_{tatABC\ 1\text{-}517}$-*lac*$^{+}$ [d]
JRE704	Δ*tatABC11* Δ*pspA162*::Cm *att*λ::pAH125::$P_{tatABC\ 1\text{-}769}$-*lac*$^{+}$ [d]
JRE708	Δ*tatABC11* Δ*pspA162*::Cm *att*λ::pAH125::$P_{tatABC\ 1\text{-}991}$-*lac*$^{+}$ [d]
JRE743	Δ*cpxR100*::Cm *att*λ::pAH125::$P_{tatABC\ 1\text{-}254}$-*lac*$^{+}$ [d]
JRE1031	Δ*cpxR100*::Cm *att*λ::pAH125::$P_{tatABC\ 1\text{-}517}$-*lac*$^{+}$ [d]
JRE744	Δ*cpxR100*::Cm *att*λ::pAH125::$P_{tatABC\ 1\text{-}769}$-*lac*$^{+}$ [d]
JRE775	Δ*cpxR100*::Cm *att*λ::pAH125::$P_{tatABC\ 1\text{-}991}$-*lac*$^{+}$ [d]
JRE745	Δ*tatABC11* Δ*cpxR100*::Cm *att*λ::pAH125::$P_{tatABC\ 1\text{-}254}$-*lac*$^{+}$ [d]
JRE1032	Δ*tatABC11* Δ*cpxR100*::Cm *att*λ::pAH125::$P_{tatABC\ 1\text{-}517}$-*lac*$^{+}$ [d]
JRE746	Δ*tatABC11* Δ*cpxR100*::Cm *att*λ::pAH125::$P_{tatABC\ 1\text{-}769}$-*lac*$^{+}$ [d]
JRE747	Δ*tatABC11* Δ*cpxR100*::Cm *att*λ::pAH125::$P_{tatABC\ 1\text{-}991}$-*lac*$^{+}$ [d]
JRE857	*att*λ::pAH125::$P_{tatABC\ 1\text{-}254}$ cpx binding mut-*lac*$^{+}$ [d]
JRE858	Δ*pspA162*::Cm *att*λ::pAH125::$P_{tatABC\ 1\text{-}254}$ cpx binding mut-*lac*$^{+}$ [d]
JRE859	Δ*tatABC11* *att*λ::pAH125::$P_{tatABC\ 1\text{-}254}$ cpx binding mut-*lac*$^{+}$ [d]
JRE860	Δ*pspA162*::Cm Δ*tatABC11* *att*λ::pAH125::$P_{tatABC\ 1\text{-}254}$ cpx binding mut-*lac*$^{+}$ [d]
JRE 1066	pTorA-mCherry-SsrA
JRE1067	Δ*tatABC11*::Kn pTorA-mCherry-SsrA
JRE1070	pJE234 *att*λ::pAH125::$P_{tatABC\ 1\text{-}991}$-*lac*$^{+}$ [d]
JRE1071	pJE235 *att*λ::pAH125::$P_{tatABC\ 1\text{-}991}$-*lac*$^{+}$ [d]
JRE1072	pJE236 *att*λ::pAH125::$P_{tatABC\ 1\text{-}991}$-*lac*$^{+}$ [d]
JRE1073	pJE216 *att*λ::pAH125::$P_{tatABC\ 1\text{-}991}$-*lac*$^{+}$ [d]
JRE1074	pJE217 *att*λ::pAH125::$P_{tatABC\ 1\text{-}991}$-*lac*$^{+}$ [d]
JRE1075	pJE218 *att*λ::pAH125::$P_{tatABC\ 1\text{-}991}$-*lac*$^{+}$ [d]
JRE1076	pBAD33 Δ*tatABC11* *att*λ::pAH125::$P_{tatABC\ 1\text{-}991}$-*lac*$^{+}$ [d]
JRE1077	pJE234 Δ*tatABC11* *att*λ::pAH125::$P_{tatABC\ 1\text{-}991}$-*lac*$^{+}$ [d]
JRE1078	pJE235 Δ*tatABC11* *att*λ::pAH125::$P_{tatABC\ 1\text{-}991}$-*lac*$^{+}$ [d]
JRE1079	pJE236 Δ*tatABC11* *att*λ::pAH125::$P_{tatABC\ 1\text{-}991}$-*lac*$^{+}$ [d]
JRE1080	pJE216 Δ*tatABC11* *att*λ::pAH125::$P_{tatABC\ 1\text{-}991}$-*lac*$^{+}$ [d]
JRE1081	pJE217 Δ*tatABC11* *att*λ::pAH125::$P_{tatABC\ 1\text{-}991}$-*lac*$^{+}$ [d]
JRE1101	pBAD33 *att*λ::pAH125::$P_{tatABC\ 1\text{-}991}$-*lac*$^{+}$ [d]
JRE1102	pJE218 Δ*tatABC11* *att*λ::pAH125::$P_{tatABC\ 1\text{-}991}$-*lac*$^{+}$ [d]

Plasmids	Relevant Characteristics	Source or Reference[b]
pKD46	*bla* P_{BAD} *gam bet exo* pSC101 oriTS	(7)
pCP20	*bla cat cI*857 P_R *flp* pSC101 oriTS	(8)
pKD3	*bla* FRT *cat* FRT PS1 PS2 oriR6K	(7)
pKD13	*bla* FRT *aph* FRT PS1 PS2 oriR6K	(7)
pKG136	*ahp* FRT *lacZY*$^{+}$ t_{his} oriR6K	(9)
pKG137	*ahp* FRT *lacZY*$^{+}$ t_{his} oriR6K	(9)
pTorA-mCherry-SsrA	pBAD24::*torA-mCherry-ssrA*	(10)
pINT-ts	Intλ	(11)
pAH125	*lacZ* t_{L3} *attP*λ oriR6K Kan t_{mgB}	(11)
pDX1	*lacZ* t_{L3} *attP*λ oriR6K *aacIV* t_{mgB}	(12)
pJE230	pAH125::$P_{tatABC\ 1\text{-}254}$-*lac*$^{+}$ [e]	
pJE231	pAH125::$P_{tatABC\ 1\text{-}517}$-*lac*$^{+}$ [e]	
pJE232	pAH125::$P_{tatABC\ 1\text{-}769}$-*lac*$^{+}$ [e]	
pJE233	pAH125::$P_{tatABC\ 1\text{-}991}$-*lac*$^{+}$ [e]	
pJE207	pBAD30::*pspA*$^{+}$	
pJE206	pBAD30::*pspF*$^{+}$	
pBA324	pBAD30::*sirA*$^{+}$	(1)

pJE216	pBAD33::*amiA*$^+$	(4)
pJE217	pBAD33::*amiC*$^+$	(4)
pJE218	pBAD33::*sufI*$^+$	(4)
pJE234	pBAD33::*cueO*$^+$	
pJE235	pBAD33::*fhuD*$^+$	
pJE236	pBAD33::*wcaM*$^+$	
pBAD30	*bla araC* P_{BAD} pACYC184 *ori*	(13)
pBAD33	*cat araC* P_{BAD} pACYC184 *ori*	(13)
pJE229	*attP*λ oriR6K *aacIV* t_{mgB} *pspF*$^+$ *pspABCDE*$^+$	

[a] All strains are isogenic derivatives of 14028 unless indicated.

[b] This study, unless otherwise noted.

[c] ATCC, American Type Culture Collection.

[d] Indicates number of bases upstream of *tatA* start codon included in the fusion.

Table S2. Oligonucleotides used in this study.

Primer Name	Sequence	Purpose
P106	CTA TCA CAG AGG AAC ATG TAT GGG TGG TAT CGT GTA GGC TGG AGC TGC TTC	Deletion of *tatABC* using pKD13
P107	CAA CCG CCC TGG CGG GCG GTT GTG TTT AGT CAT TCC GGG GAT CCG TCG ACC	Deletion of *tatABC* using pKD13
P169	CGC GTA TAA ATG ACT GTT CGG CAT CCA GCG ATA TGA ATA TCC TCC	Deletion of *baeR* using pKD3/4
P170	CCC ATT GAT GAA AAC ACG CCG CGC ATT TTG CGT AGG CTG GAG CTG C	Deletion of *baeR* using pKD3/4
P207	CTG AAT TTA AAG ATA ACC TGC TCG GCG AGG GTA GGC TGG AGC TGC	Deletion of *pspF* using pKD3/4
P208	CTG GTG GTA GGT GAG CGC CAG CAA ATC GGC ATA TGA ATA TCC TCC	Deletion of *pspF* using pKD3/4
P231	GCC GAC ATC GTG AAC GCC AAT ATC AAT GCG GTA GGC TGG AGC TGC	Deletion of *pspA* using pKD3/4
P232	CAA CTG CGC CAG CTG CTC GCT GAT TTC ATC ATA TGA ATA TCC TCC	Deletion of *pspA* using pKD3/4
P211	GAT CGA ATT CCA GAA CAT TAT GTG AGG ATT G	Cloning of *pspA* into pBAD30
P212	CTA GTC TAG ACC ATT GTC ATT ATT GAT TAT C	Cloning of *pspA* into pBAD30
P213	GAT CGA ATT CGT GGC GAT TTT CAT CAT GGC TG	Cloning of *pspF* into pBAD30
P214	CTA GTC TAG AGA GAA ATA GCT AAA GCT GAT GC	Cloning of *pspF* into pBAD30
P301	GAT CGG TAC CGT CGG CGG CGT AGT TGT C	Cloning of 250 bp *tatABC* promoter fragment
P303	GAT CGA ATT CAC ATG TTC CTC TGT GAT AG	Cloning of all *tatABC* promoter fragments
P304	GAT CGG TAC CCA TGC GGA CAT GCA TCC GG	Cloning of 1000 bp *tatABC* promoter fragment

P305	GAT CGG TAC CGA GTT TGC CAT CCG CAC CG	Cloning of 750 bp *tatABC* promoter fragment
P306	GAT CGG TAC CCC GGC TCT CAC GCG GGC TC	Cloning of 500 bp *tatABC* promoter fragment
P344	GAT CCG ATC GCA TAC CTG TGA CGG AAG ATC	Cloning *cat* into pAT6
P397	GAT CCG ATC GCA CTT ATT CAG GCG TAG C	Cloning *cat* into pAT6
P278	GAT CGG TAC CGT TCA TTA TAA GGA AAT G	Cloning of *cueO* into pBAD33
P130	ACG ATC TAG ATC AGA CCG TAA ATC CTA	Cloning of *cueO* into pBAD33
P353	GAT CGG TAC CGA TGG GTA TCC TTC CGC A	Cloning of *fhuD* into pBAD33
P354	GAT CTC TAG ACA CGT TTT CTG CTC ACG C	Cloning of *fhuD* into pBAD33
P355	GAT CGG TAC CGC CAG CCT GCT ACA AAC G	Cloning of *wcaM* into pBAD33
P356	GAT CTC TAG AGC GGT ACT TAC CCT CCC	Cloning of *wcaM* into pBAD33
P395	GAT CGC ATG CCG GTT AGT ATG ATT GAG C	Cloning *psp* region into pDX1
P396	GAT CGC TAG CGG CGT AAA TGT CGT CAA TTA G	Cloning *psp* region into pDX1

SUPPLEMENTAL REFERENCES

1. Ahmer BMM, Van Reeuwijk J, Watson PR, Wallis TS, Heffron F. 1999. *Salmonella* SirA is a global regulator of genes mediating enteropathogenesis. *Molecular Microbiology* 31:971–982.

2. Golubeva YA, Sadik AY, Ellermeier JR, Slauch JM. 2012. Integrating global regulatory input into the *Salmonella* pathogenicity Island 1 type III secretion system. *Genetics* 190:79–90.

3. Wang Q, Zhao Y, McClelland M, Harshey RM. 2007. The RcsCDB signaling system and swarming motility in *Salmonella enterica* serovar typhimurium: dual regulation of flagellar and SPI-2 virulence genes. *Journal of Bacteriology* 189:8447–57.

4. Brauer AM, Rogers AR, Ellermeier JR. 2021. Twin-arginine translocation (Tat) mutants in *Salmonella enterica* serovar Typhimurium have increased susceptibility to cell wall targeting antibiotics. *FEMS Microbes* 2.

5. Ellermeier CD, Slauch JM. 2004. RtsA coordinately regulates DsbA and the *Salmonella* pathogenicity island 1 type III secretion system. *Journal of Bacteriology* 186:68–79.

6. Golubeva YA, Ellermeier JR, Cott Chubiz JE, Slauch JM. 2016. Intestinal Long-Chain Fatty Acids Act as a Direct Signal To Modulate Expression of the *Salmonella* Pathogenicity Island 1 Type III Secretion System. *mBio* 7:e02170–15.

7. Datsenko KA, Wanner BL. 2000. One-step inactivation of chromosomal genes in *Escherichia coli* K-12 using PCR products. *Proceedings of the National Academy of Sciences* 97:6640–6645.

8. Cherepanov PP, Wackernagel W. 1995. Gene disruption in *Escherichia coli*: TcR and KmR cassettes with the option of Flp-catalyzed excision of the antibiotic-resistance determinant. *Gene* 158:9–14.

9. Ellermeier CD, Janakiraman A, Slauch JM. 2002. Construction of targeted single copy *lac* fusions using λ Red and FLP-mediated site-specific recombination in bacteria. *Gene* 290:153–161.

10. Bageshwar UK, VerPlank L, Baker D, Dong W, Hamsanathan S, Whitaker N, Sacchettini JC, Musser SM. 2016. High throughput screen for *Escherichia coli* twin arginine translocation (Tat) inhibitors. *PLoS One* 11.

11. Haldimann A, Wanner BL. 2001. Conditional-replication, integration, excision, and retrieval plasmid-host systems for gene structure-function studies of bacteria. *Journal of Bacteriology* 183:6384–6393.

12. Ellermeier JR, Slauch JM. 2008. Fur regulates expression of the *Salmonella* pathogenicity island 1 type III secretion system through HilD. *Journal of Bacteriology* 190:476–486.

13. Guzman L-M, Belin D, Carson MJ, Beckwith J. 1995. Tight Regulation, Modulation, and High-Level Expression by Vectors Containing the Arabinose P_{BAD} Promoter. *Journal of Bacteriology* 177:4121–4130.
